# Association between chronic kidney disease and heart failure across the ejection fraction spectrum: a retrospective case-control study from the Swedish Heart Failure Registry

**DOI:** 10.1093/eschf/xvag196

**Published:** 2026-07-19

**Authors:** Valeria Valente, Lina Benson, Carin Corovic Cabrera, Raffaele Scorza, Felix Lindberg, Ida Haugen Löfman, Michael Melin, Lars H Lund, Giulia Ferrannini, Gianluigi Savarese

**Affiliations:** Department of Clinical Science and Education, Södersjukhuset, Karolinska Institutet, Stockholm South Hospital, Sjukhusbacken 10, Stockholm 118 83, Sweden; Department of Clinical Science and Education, Södersjukhuset, Karolinska Institutet, Stockholm South Hospital, Sjukhusbacken 10, Stockholm 118 83, Sweden; Division of Cardiology, Department of Medicine, Karolinska Institutet, Stockholm, Sweden; Department of Clinical Science and Education, Södersjukhuset, Karolinska Institutet, Stockholm South Hospital, Sjukhusbacken 10, Stockholm 118 83, Sweden; Department of Clinical Science and Education, Södersjukhuset, Karolinska Institutet, Stockholm South Hospital, Sjukhusbacken 10, Stockholm 118 83, Sweden; Department of Clinical Science and Education, Södersjukhuset, Karolinska Institutet, Stockholm South Hospital, Sjukhusbacken 10, Stockholm 118 83, Sweden; Division of Cardiology, Department of Medicine, Karolinska Institutet, Stockholm, Sweden; Department of Clinical Science and Education, Södersjukhuset, Karolinska Institutet, Stockholm South Hospital, Sjukhusbacken 10, Stockholm 118 83, Sweden; Division of Cardiology, Department of Medicine, Karolinska Institutet, Stockholm, Sweden; Division of Cardiology, Department of Medicine, Karolinska Institutet, Stockholm, Sweden; Internal Medicine Unit, Södertälje Hospital, Södertälje, Sweden; Department of Clinical Science and Education, Södersjukhuset, Karolinska Institutet, Stockholm South Hospital, Sjukhusbacken 10, Stockholm 118 83, Sweden

**Keywords:** Chronic kidney disease, Heart failure, Phenotypes, Registry, SwedeHF

## Abstract

**Background and Aims:**

Heart failure (HF) and chronic kidney disease (CKD) are prevalent and often co-exist. Whether CKD is preferentially associated with a specific HF ejection fraction (EF) phenotype remains unclear. We aimed to investigate the association between CKD and new-onset HF across the EF spectrum and according to CKD severity.

**Methods:**

Patients with a first HF diagnosis from the Swedish HF Registry (2005–2021) were matched 1:1 by sex, year of birth, and county to controls without HF. In separate analyses, CKD was defined according to (i) ICD-10 codes in both HF patients and controls; (ii) an estimated glomerular filtration rate (eGFR) < 60 ml/min/1.73m^2^ in HF patients. Renal function was further stratified into the KDIGO categories G2−G5, i.e. G2:60–89;G3a:45–59; G3b:30–44; G4:15–29; G5 < 15 ml/min/1.73 m^2^ in eGFR-defined CKD analyses.

**Results:**

51 848 HF patients (20% HF with preserved EF [HFpEF], 23% HF with mildly reduced EF [HFmrEF], 57% HF with reduced EF [HFrEF]) and 51 848 controls were considered. CKD was associated with ∼two-fold higher odds of HF, particularly HFpEF, compared with HFmrEF and HFrEF vs non-HF (ICD-10 definition: odds ratio [OR]2.46, 1.30, and 1.52, respectively; *P*-interaction=0.001) and with 8% higher odds of HFpEF vs HFrEF when defined by eGFR in the HF population. Across KDIGO stages, with G2 as reference, G3b–G5 were more likely associated with HFpEF vs HFrEF, G3a had similar associations with HFpEF and HFrEF, and G3a–G3b were less likely associated with HFmrEF vs. HFrEF.

**Conclusions:**

CKD was independently associated with new-onset HF, especially HFpEF in moderate to advanced CKD, and both HFpEF and HFrEF in mild CKD. Targeting CKD may be crucial to prevent HF.

## Graphical Abstract

**Graphical Abstract:**
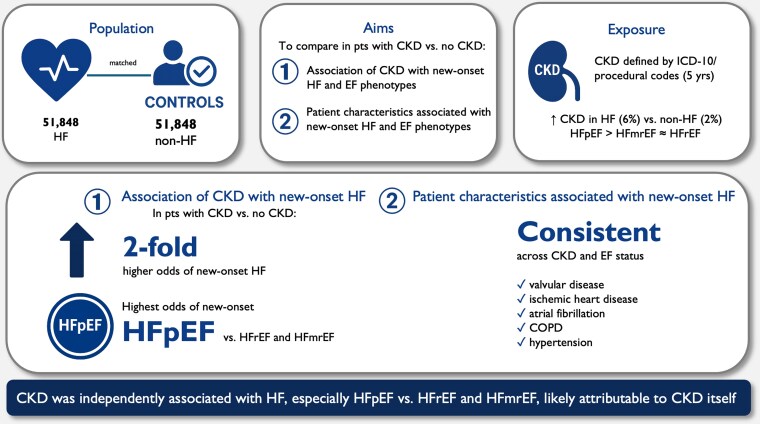
HF, heart failure; CKD, chronic kidney disease; eGFR, estimated glomerular filtration rate; EF, ejection fraction; HFrEF, heart failure with reduced ejection fraction; HFmrEF, heart failure with mildly reduced ejection fraction; HFpEF, heart failure with preserved ejection fraction; ICD-10, International Classification of Diseases, 10th Revision; COPD, chronic obstructive pulmonary disease.

## Introduction

Heart failure (HF) represents a growing public health concern, as nearly one in four individuals develop HF during their lifetime.^1^ Preventing HF onset is therefore critical and relies on identifying and mitigating the effect of its risk factors across diverse populations.^2^

Chronic kidney disease (CKD) frequently coexists with HF.^3–5^ Approximately half of patients with HF have concomitant CKD, while HF affects 17% to 50% of individuals with CKD, depending on age and specific renal function.^3–5^ Their overlap complicates diagnosis, risk stratification, and management of both conditions, and is associated with worse outcomes.^6–9^

Despite accumulating evidence linking CKD to increased HF risk, longitudinal studies have yielded conflicting results.^3,10,11^ This inconsistency may stem from methodological discrepancies in CKD definition across studies, such as considering single measurements of estimated glomerular filtration rate (eGFR) or serum creatinine with variable thresholds, and diverse biomarkers capturing distinct stages and clinical presentations of renal dysfunction.^3,10,11^ Moreover, part of this inconsistency may also derive from differences in HF ascertainment across investigations, as previous studies variably captured hospitalized or outpatient HF diagnoses, relied on administrative codes or adjudicated events, and applied heterogeneous diagnostic criteria before the Universal Definition of HF was released in 2021.^3,10–12^ Significant heterogeneity in study design, patient selection, and statistical power may have further introduced bias and hindered comparability across studies.^3,10,11^

Controversy also persists regarding whether patients with CKD are more likely to develop HF with preserved (HFpEF) vs. reduced ejection fraction (HFrEF).^3,10,11^ HF with mildly reduced EF (HFmrEF), previously considered a ‘grey zone’ between HFrEF and HFpEF, is now recognized as a HF phenotype that more closely resembles HFrEF than HFpEF, particularly with respect to ischaemic aetiology and response to HFrEF therapies.^13,14^ Nevertheless, HFmrEF has often been excluded, grouped with either HFrEF or HFpEF, or inconsistently defined in prior studies, further preventing robust comparisons across EF phenotypes.^2,4,15,16^ Moreover, it remains unclear whether the association between CKD and EF phenotypes differs according to CKD severity.^17,18^

While several risk factors for HF are known, it remains largely unexplored whether patient characteristics associated with new-onset HF, and specifically HFpEF or HFmrEF or HFrEF, differ in those with and without CKD.^2,19^

To address these gaps, the current study aimed to explore (i) the association between CKD and new-onset HF across the EF spectrum; (ii) whether this association differed according to CKD severity; and (iii) patient characteristics independently linked with new-onset HF and HFpEF vs HFrEF and HFmrEF according to CKD status.

## Methods

This study had a retrospective, matched case-control design. Patients with new-onset HF from the Swedish Heart Failure Registry (SwedeHF) were matched 1:1 by sex, year of birth, and county of residence with a non-HF cohort randomly selected from the general Swedish population through Statistics Sweden.

### Data sources

The study cohort was derived from SwedeHF and linked to the National Patient Register, the Cause of Death Register, the Swedish Prescribed Drug Register, and Statistics Sweden. Detailed information on these data sources is provided in the Supplementary Table S1*B*.

### Definitions

The index date was the date of discharge for inpatients or the date of visit for outpatients related to the registration in SwedeHF. Matched non-HF individuals were assigned the same index date as their matched HF case.

HFpEF was defined as EF ≥ 50%, HFmrEF as EF 40%–49%, HFrEF as EF < 40%. This classification is slightly different as compared with the Universal Definition and recent European and American guidelines due to EF being recorded in SwedeHF as a categorical variable in the majority of patients, which may have led to misclassification of patients with EF 40% as HFmrEF rather than HFrEF.^6–8,12^

Two definitions of CKD were applied in the current study. International Statistical Classification of Diseases and Related Health Problems, 10th Revision (ICD-10) codes-based CKD was defined by the presence of at least one registration of the following ICD-10 or procedural codes N18, N18.1–5, N19, Z491, Z492, DR014, DR016, DR020, DR012, DR013, DR024, TJA33, TJA35, Y841, T824, Z490, Z992, Z492, DR055, DR056, DR060, DR061, QF006, I120, I130, I131, I132, N085, M103, E122, E112, E132, E102, E142, R944, N02, N06, N25, N11, N14, N15, N16, Q611–3, Q60-Q64, N03, N04, N06, N07, N08, K767, N250 in the National Patient Register within the last five years prior to the index date (Supplementary Table S1*A*). Since the ICD-10 diagnosis for CKD might be under-reported or reported only in more severe cases, in separate analyses including only the HF population, as laboratory data were not available in the controls without HF, CKD was defined according to the estimated glomerular filtration rate (eGFR) < 60 ml/min/1.73 m^2^ calculated using the 2021 Chronic Kidney Disease Epidemiology Collaboration (2021 CKD-EPI) equation. Serum creatinine levels were collected prior to (close to) discharge for inpatients or scheduled visits for outpatients. If creatinine was not measured or the latest value was older than one month, it was reported as unknown.

The definitions of all the variables considered in this study are provided in Supplementary Table S1*B*.

### Patients

The full selection process of the study population is reported in detail in Supplementary Figure S1. In the HF population, only patients with an HF duration of <6 months, i.e. new-onset HF, registered in SwedeHF between 1 December 2005 and 31 December 2021, without missing entries for EF and alive at discharge, were considered. If an HF patient had multiple registrations, only the first one was selected.

The control cohort without HF cohort was derived through Statistics Sweden, and included individuals without any HF diagnoses (ICD-10 codes I110, I130, I132, I255, I420, I423, 1425–9, I43, I50, J81, K761, R570, ICD-9: 414W, 425E-H, 425W, 425X, 428) in the National Patient Register and without any registration in SwedeHF, up to the end of the year corresponding to the registration date of the matched HF case in SwedeHF.

Patients with missing socioeconomic records, or unmatched HF and non-HF patients, were excluded from the analysis. The exclusion of a patient with HF from SwedeHF due to the application of selection criteria resulted in the removal of the corresponding non-HF matched control.

For the HF cohort with available eGFR measurements, the same inclusion criteria were applied, with the additional exclusion of patients without documented eGFR measurements or with ICD-10/procedural codes for acute kidney disease (N17, N00, N01, N10, N12, N13.0–N13.6, N15.1, N20, I82.3, N28.0, N28.0, DR015, DR023, TKA30) within the last year before the index date.

### Statistical analysis

Patient characteristics were presented as frequencies and percentages for categorical variables, and as medians with interquartile ranges for continuous variables, and their differences across the groups with vs. without CKD were assessed by chi-square test for categorical variables, and Kruskal-Wallis test for continuous variables.

The association between ICD-defined CKD and new-onset HF was assessed by univariable and multivariable conditional logistic regression models stratified by matching pairs. An interaction term between CKD and EF/case/control category was included to detect eventual differences in the association between CKD and new-onset HFpEF, HFmrEF, and HFrEF vs. non-HF. Results were reported as odds ratios (OR) with 95% confidence intervals (CI).

To investigate whether patient characteristics linked with new-onset HF differed according to CKD status, we performed multiple multivariable conditional logistic regression models stratified by matching pairs with HF as the dependent variable and including the interaction terms between each covariate/patient characteristic and CKD. To assess whether these results differed in HFpEF and HFmrEF vs HFrEF, multivariable multinomial logistic regression models were then fitted, including an interaction term between each patient characteristic and CKD. Only variables significantly associated with new-onset HF in the conditional logistic regression models were tested in the multinomial regression models. Results were summarized as OR with 95% CI. Covariates included in these models are marked with the letter a in *Tables 1* and *2*, and had no missing data.

**Table 1:** Baseline characteristics of patients with new-onset HF and matched non-HF controls according to ICD-defined CKD status

Characteristics	Matched non-HF controls			HF		
	No CKD	CKD	*P-value*	No CKD	CKD	*P-value*
*N* (%)	50 914 (98)	934 (2)		48 924 (94)	2924 (6)	
**Demographics**						
Sex male, *n* (%)	31 779 (62)	701 (75)	<.001	30 501 (62)	1979 (68)	<.001
Age, years, median [IQR]	74 [64, 81]	79 [73, 84]	<.001	73 [64, 81]	76 [68, 83]	<.001
**History and comorbidities**						
Alcohol, *n* (%)	750 (1)	30 (3)	<.001	1696 (3)	85 (3)	.11
Diabetes, *n* (%)^a^	3834 (8)	341 (37)	<.001	9995 (20)	1278 (44)	<.001
Peripheral artery disease, *n* (%)^a^	1204 (2)	105 (11)	<.001	3283 (7)	470 (16)	<.001
Hypertension, *n* (%)^a^	10 146 (20)	677 (72)	<.001	29 740 (61)	2462 (84)	<.001
Stroke/TIA, *n* (%)^a^	4760 (9)	199 (21)	<.001	6397 (13)	589 (20)	<.001
Ischemic heart disease, *n* (%)^a^	6229 (12)	222 (24)	<.001	21 186 (43)	1546 (53)	<.001
Atrial fibrillation, *n* (%)^a^	3200 (6)	141 (15)	<.001	23 126 (47)	1310 (45)	.009
Malignant cancer within 3 years, *n* (%)^a^	5268 (10)	209 (22)	<.001	5764 (12)	455 (16)	<.001
Valvular disease, *n* (%)^a^	962 (2)	53 (6)	<.001	7965 (16)	542 (19)	.001
COPD, *n* (%)^a^	1302 (3)	74 (8)	<.001	5063 (10)	349 (12)	.006
Liver disease, *n* (%)^a^	349 (1)	22 (2)	<.001	921 (2)	100 (3)	<.001
Musculoskeletal/connective tissue disease within 3 years, *n* (%)^a^	10 039 (20)	346 (37)	<.001	13 783 (28)	1123 (38)	<.001
Dementia, *n* (%)	1615 (3)	48 (5)	<.001	616 (1)	42 (1)	.41
Depression, *n* (%)	1138 (2)	29 (3)	.076	1739 (4)	131 (4)	.009
CCI, *n* (%)						
0–1	37 201 (73)	35 (4)	<.001	18 375 (38)	54 (2)	<.001
2–3	10 036 (20)	355 (38)		19 260 (39)	584 (20)	
4–7	3035 (6)	444 (48)		9380 (19)	1823 (62)	
≥8	642 (1)	100 (11)		1909 (4)	463 (16)	
**Medications**						
RASi/ARNI, *n* (%)	15 198 (30)	469 (50)	<.001	44 251 (90)	2339 (80)	<.001
MRA, *n* (%)	771 (2)	20 (2)	.12	17 847 (36)	740 (25)	<.001
Digoxin, *n* (%)	465 (1)	10 (1)	.62	6851 (14)	212 (7)	<.001
Diuretic, *n* (%)	10 281 (20)	479 (51)	<.001	35 471 (73)	2526 (86)	<.001
Nitrate, *n* (%)	2061 (4)	63 (7)	<.001	12 869 (26)	889 (30)	<.001
Platelet inhibitor, *n* (%)	11 341 (22)	390 (42)	<.001	23 315 (48)	1636 (56)	<.001
Statin, *n* (%)	12 275 (24)	448 (48)	<.001	23 984 (49)	1750 (60)	<.001
Oral anticoagulant, *n* (%)	3786 (7)	146 (16)	<.001	22 755 (47)	1208 (41)	<.001
Beta-blockers, *n* (%)	12 208 (24)	470 (50)	<.001	43 985 (90)	2623 (90)	.73
Calcium channel blockers, *n* (%)	9397 (18)	404 (43)	<.001	10 946 (22)	1406 (48)	<.001
SGLT2i, *n* (%)	288 (1)	5 (1)	.90	1244 (3)	53 (2)	.014
Education, *n* (%)^a^						
Compulsory school	18 329 (36)	389 (42)	<.001	19 844 (41)	1289 (44)	<.001
Secondary school	20 192 (40)	353 (38)		20 162 (41)	1143 (39)	
University	12 393 (24)	192 (21)		8918 (18)	492 (17)	
Disposable income, above median within year, *n* (%)^a^	27 071 (53)	459 (49)	.015	23 072 (47)	1270 (43)	<.001
Family type, Living alone, *n* (%)^a^	21 438 (42)	416 (45)	.14	22 756 (47)	1369 (47)	.75

^a^Variables included in the adjusted models;

*P*-values compare patients with versus without CKD within each group.

Abbreviations: HF, heart failure; CKD, chronic kidney disease; SwedeHF, Swedish Heart Failure Registry; IQR, interquartile range; TIA, transient ischaemic attack; COPD, chronic obstructive pulmonary disease; CCI, Charlson comorbidity index; RASi, renin-angiotensin system inhibitor; ARNI, angiotensin receptor neprilysin inhibitor; MRA, mineralocorticoid receptor antagonists; SGLT2i, sodium/glucose cotransporter-2 inhibitors.

**Table 2:** Baseline characteristics of patients with new-onset HF across the EF spectrum and matched non-HF controls according to ICD-defined CKD status

Characteristics		Matched non-HF controls			HFrEF			Matched non-HF controls			HFmrEF			Matched non-HF controls			HFpEF		
		no CKD	CKD	*P-value*	no CKD	CKD	*P-value*	no CKD	CKD	*P-value*	no CKD	CKD	*P-value*	no CKD	CKD	*P-value*	no CKD	CKD	*P-value*
*N* (%)		28 914 (98)	485 (2)		27 952 (95)	1447 (5)		11 547 (98)	248 (2)		11 166 (95)	629 (5)		10 453 (98)	201 (2)		9806 (92)	848 (8)	
**Demographics**																			
Sex Male, *n* (%)		19 958 (69)	386 (80)	<.001	19 270 (69)	1074 (74)	<.001	7054 (61)	193 (78)	<.001	6836 (61)	411 (65)	.039	4767 (46)	122 (61)	<.001	4395 (45)	494 (58)	<.001
Age, years, median [IQR]		71 [62, 79]	78 [72, 83]	<.001	71 [62, 79]	75 [66, 82]	<.001	74 [65, 81]	81 [75, 84]	<.001	74 [65, 81]	76 [67, 82]	<.001	79 [72, 84]	81 [75, 85]	.001	78 [71, 84]	79 [72, 84]	.48
**History and comorbidities**																			
Alcohol, *n* (%)		473 (2)	15 (3)	.013	1147 (4)	46 (3)	.082	179 (2)	7 (3)	.11	292 (3)	19 (3)	.54	98 (1)	8 (4)	<.001	257 (3)	20 (2)	.65
Diabetes, *n* (%)^a^		2096 (7)	193 (40)	<.001	5454 (20)	553 (38)	<.001	888 (8)	82 (33)	<.001	2203 (20)	302 (48)	<.001	850 (8)	66 (33)	<.001	2338 (24)	423 (50)	<.001
Peripheral artery disease, *n* (%)^a^		621 (2)	53 (11)	<.001	1722 (6)	230 (16)	<.001	304 (3)	34 (14)	<.001	752 (7)	101 (16)	<.001	279 (3)	18 (9)	<.001	809 (8)	139 (16)	<.001
Hypertension, *n* (%)^a^		5307 (18)	346 (71)	<.001	15 232 (54)	1153 (80)	<.001	2414 (21)	184 (74)	<.001	7136 (64)	537 (85)	<.001	2425 (23)	147 (73)	<.001	7372 (75)	772 (91)	<.001
Stroke/TIA, *n* (%)^a^		2428 (8)	100 (21)	<.001	3285 (12)	268 (19)	<.001	1132 (10)	64 (26)	<.001	1478 (13)	128 (20)	<.001	1200 (11)	35 (17)	.009	1634 (17)	193 (23)	<.001
Ischaemic heart disease, *n* (%)^a^		3353 (12)	106 (22)	<.001	11 789 (42)	764 (53)	<.001	1418 (12)	66 (27)	<.001	5442 (49)	366 (58)	<.001	1458 (14)	50 (25)	<.001	3955 (40)	416 (49)	<.001
Atrial fibrillation, *n* (%)^a^		1691 (6)	75 (15)	<.001	12 010 (43)	610 (42)	.54	775 (7)	35 (14)	<.001	5416 (49)	294 (47)	.39	734 (7)	31 (15)	<.001	5700 (58)	406 (48)	<.001
Malignant cancer within 3 years, *n* (%)^a^		2893 (10)	109 (22)	<.001	2986 (11)	212 (15)	<.001	1200 (10)	55 (22)	<.001	1415 (13)	90 (14)	.23	1175 (11)	45 (22)	<.001	1363 (14)	153 (18)	<.001
Valvular disease, *n* (%)^a^		510 (2)	18 (4)	.001	3529 (13)	225 (16)	.001	230 (2)	23 (9)	<.001	1964 (18)	119 (19)	.39	222 (2)	12 (6)	<.001	2472 (25)	198 (23)	.23
COPD, *n* (%)^a^		668 (2)	27 (6)	<.001	2588 (9)	155 (11)	.064	321 (3)	24 (10)	<.001	1102 (10)	71 (11)	.25	313 (3)	23 (11)	<.001	1373 (14)	123 (15)	.69
Liver disease, *n* (%)^a^		204 (1)	10 (2)	<.001	536 (2)	55 (4)	<.001	86 (1)	8 (3)	<.001	171 (2)	19 (3)	.004	59 (1)	4 (2)	.009	214 (2)	26 (3)	.096
Musculoskeletal/connective tissue disease within 3 years, *n* (%)^a^		5475 (19)	169 (35)	<.001	6835 (24)	522 (36)	<.001	2328 (20)	89 (36)	<.001	3387 (30)	247 (39)	<.001	2236 (21)	88 (44)	<.001	3561 (36)	354 (42)	.002
Dementia, *n* (%)		714 (2)	20 (4)	.021	297 (1)	21 (1)	.16	395 (3)	12 (5)	.23	117 (1)	14 (2)	.006	506 (5)	16 (8)	.042	202 (2)	7 (1)	.013
Depression, *n* (%)		583 (2)	12 (2)	.48	996 (4)	71 (5)	.008	285 (2)	7 (3)	.72	347 (3)	27 (4)	.099	270 (3)	10 (5)	.036	396 (4)	33 (4)	.83
CCI, *n* (%)	**0–1**	21 651 (75)	21 (4)	<.001	11 130 (40)	33 (2)	<.001	8349 (72)	9 (4)	<.001	4069 (36)	10 (2)	<.001	7201 (69)	5 (2)	<.001	3176 (32)	11 (1)	<.001
	**2–3**	5349 (18)	189 (39)		10 928 (39)	309 (21)		2300 (20)	87 (35)		4494 (40)	121 (19)		2387 (23)	79 (39)		3838 (39)	154 (18)	
	**4–7**	1586 (5)	226 (47)		4944 (18)	899 (62)		739 (6)	127 (51)		2130 (19)	396 (63)		710 (7)	91 (45)		2306 (24)	528 (62)	
	**≥8**	328 (1)	49 (10)		950 (3)	206 (14)		159 (1)	25 (10)		473 (4)	102 (16)		155 (1)	26 (13)		486 (5)	155 (18)	
**Medications**																			
RASi/ARNI, *n* (%)		8327 (29)	260 (54)	<.001	26 524 (95)	1218 (84)	<.001	3564 (31)	112 (45)	<.001	10 099 (90)	510 (81)	<.001	3307 (32)	97 (48)	<.001	7628 (78)	611 (72)	<.001
MRA, *n* (%)		362 (1)	8 (2)	.44	11 619 (42)	393 (27)	<.001	204 (2)	5 (2)	.77	2929 (26)	124 (20)	<.001	205 (2)	7 (3)	.13	3299 (34)	223 (26)	<.001
Digoxin, *n* (%)		223 (1)	5 (1)	.52	3956 (14)	114 (8)	<.001	109 (1)	2 (1)	.82	1371 (12)	33 (5)	<.001	133 (1)	3 (1)	.78	1524 (16)	65 (8)	<.001
Diuretic, *n* (%)		5292 (18)	251 (52)	<.001	20 454 (73)	1218 (84)	<.001	2392 (21)	125 (50)	<.001	7030 (63)	530 (84)	<.001	2597 (25)	103 (51)	<.001	7987 (81)	778 (92)	<.001
Nitrate, *n* (%)		1036 (4)	30 (6)	.002	7287 (26)	444 (31)	<.001	471 (4)	12 (5)	.55	3337 (30)	209 (33)	.075	554 (5)	21 (10)	.001	2245 (23)	236 (28)	.001
Platelet inhibitor, *n* (%)		5989 (21)	193 (40)	<.001	13 648 (49)	831 (57)	<.001	2576 (22)	118 (48)	<.001	5551 (50)	363 (58)	<.001	2776 (27)	79 (39)	<.001	4116 (42)	442 (52)	<.001
Statin, *n* (%)		6772 (23)	234 (48)	<.001	13 709 (49)	837 (58)	<.001	2893 (25)	120 (48)	<.001	5907 (53)	398 (63)	<.001	2610 (25)	94 (47)	<.001	4368 (45)	515 (61)	<.001
Oral anticoagulant, *n* (%)		1991 (7)	79 (16)	<.001	12 665 (45)	588 (41)	<.001	947 (8)	37 (15)	<.001	5124 (46)	274 (44)	.25	848 (8)	30 (15)	<.001	4966 (51)	346 (41)	<.001
Beta-blocker, *n* (%)		6587 (23)	240 (49)	<.001	26 035 (93)	1324 (91)	.017	2733 (24)	118 (48)	<.001	9792 (88)	563 (90)	.18	2888 (28)	112 (56)	<.001	8158 (83)	736 (87)	.007
Calcium channel blockers, *n* (%)		5013 (17)	222 (46)	<.001	4845 (17)	573 (40)	<.001	2229 (19)	101 (41)	<.001	2684 (24)	316 (50)	<.001	2155 (21)	81 (40)	<.001	3417 (35)	517 (61)	<.001
SGLT2i, *n* (%)		170 (1)	3 (1)	.93	908 (3)	37 (3)	.15	87 (1)	1 (<1)	.53	226 (2)	7 (1)	.01	31 (<1)	1 (<1)	.61	110 (1)	9 (1)	.87
**Socio-economic characteristics**																			
Education, *n* (%)^a^	**Compulsory school**	9733 (34)	199 (41)	.003	10 863 (39)	608 (42)	.056	4173 (36)	108 (44)	.003	4435 (40)	277 (44)	.092	4423 (42)	82 (41)	.68	4546 (46)	404 (48)	.59
	**Secondary school**	11 852 (41)	181 (37)		11 973 (43)	588 (41)		4541 (39)	101 (41)		4581 (41)	243 (39)		3799 (36)	71 (35)		3608 (37)	312 (37)	
	**University**	7329 (25)	105 (22)		5116 (18)	251 (17)		2833 (25)	39 (16)		2150 (19)	109 (17)		2231 (21)	48 (24)		1652 (17)	132 (16)	
Disposable income, Above median within year, *n* (%)^a^		16 576 (57)	260 (54)	.10	13 970 (50)	671 (46)	.007	6037 (52)	109 (44)	.009	5435 (49)	262 (42)	<.001	4458 (43)	90 (45)	.55	3667 (37)	337 (40)	.18
Family type, Living alone, *n* (%)^a^		11 333 (39)		.022	12 712 (45)	660 (46)	.92	4938 (43)	107 (43)	.90	4907 (44)	290 (46)	.29	5167 (49)	94 (47)	.45	5137 (52)	419 (49)	.096

^a^Variables included in the adjusted models;

*P*-values compare patients with versus without CKD within each EF/control group.

Abbreviations: HF, heart failure; CKD, chronic kidney disease; HFrEF, heart failure with reduced ejection fraction; HFmrEF, heart failure with mid-range or mildly reduced ejection fraction; HFpEF, heart failure with preserved ejection fraction; IQR, interquartile range; TIA, transient ischaemic attack; COPD, chronic obstructive pulmonary disease; CCI, Charlson comorbidity index; RASi, renin-angiotensin system inhibitor; ARNI, angiotensin receptor neprilysin inhibitor; MRA, mineralocorticoid receptor antagonists; SGLT2i, sodium/glucose cotransporter-2 inhibitors.

In patients with HF where we had eGFR measurements, we fitted univariable and multivariable multinomial logistic regression models including the variables labelled with letter a in *Table 1* with CKD defined as eGFR of <60 ml/min/1.73 m^2^ to assess potential differences in the association between CKD and HFpEF vs HFmrEF vs HFrEF. Univariable and multivariable multinomial logistic regression models were also fitted to test whether associations between CKD and HFpEF, HFmrEF, and HFrEF differed by severity of renal impairment. Renal function was further stratified according to the kidney disease: Improving Global Outcomes (KDIGO) eGFR categories: G2 (60–89 ml/min/1.73 m^2^, reference), G3a (45–59), G3b (30–44), G4 (15–29), and G5 (<15). Patients with eGFR ≥90 ml/min/1.73 m^2^ were not included in stratified analyses, as evidence of kidney damage (e.g. proteinuria or haematuria) was unavailable to confirm CKD stage G1. Missing data in the SwedeHF population and the proportion of missing values for each variable are reported in *Table 3*. No variable included in the multivariable models contained missing values.

**Table 3:** Baseline characteristics of patients with new-onset HF according to EF phenotype and eGFR-defined CKD status

Characteristics		HF			HFrEF			HFmrEF			HFpEF			Missing (%)
		eGFR ≥ 60	eGFR<60	*P-value*	eGFR ≥ 60	eGFR<60	*P-value*	eGFR ≥ 60	eGFR<60	*P-value*	eGFR ≥ 60	eGFR<60	*P-value*	
*N* (%)		34 536 (70)	14 913 (30)		20 652 (73)	7512 (27)		7921 (71)	3308 (29)		5963 (59)	4093 (41)		
**Demographics**														
Sex male, *n* (%)^a^		23 032 (67)	7909 (53)	<.001	14 892 (72)	4548 (61)	<.001	5163 (65)	1736 (52)	<.001	2977 (50)	1625 (40)	<.001	
Age, years, median [IQR]^a^		70 [61, 78]	80 [74, 85]	<.001	68 [59, 76]	78 [72, 84]	<.001	71 [62, 78]	80 [74, 85]	<.001	76 [68, 82]	82 [76, 86]	<.001	
**History and comorbidities**														
Alcohol, *n* (%)		1412 (4)	259 (2)	<.001	969 (5)	157 (2)	<.001	244 (3)	42 (1)	<.001	199 (3)	60 (1)	<.001	
Diabetes, *n* (%)^a^		6372 (18)	4176 (28)	<.001	3685 (18)	1960 (27)	<.001	1396 (18)	943 (29)	<.001	1291 (22)	1273 (32)	<.001	
Peripheral artery disease, *n* (%)^a^		1954 (6)	1486 (10)	<.001	1062 (5)	723 (10)	<.001	455 (6)	319 (10)	<.001	437 (7)	444 (11)	<.001	
Hypertension, *n* (%)^a^		19 292 (56)	11 197 (75)	<.001	10 392 (50)	5181 (69)	<.001	4660 (59)	2591 (78)	<.001	4240 (71)	3425 (84)	<.001	
Stroke/TIA, *n* (%)^a^		3851 (11)	2747 (18)	<.001	2051 (10)	1309 (17)	<.001	904 (11)	611 (18)	<.001	896 (15)	827 (20)	<.001	
Ischaemic heart disease, *n* (%)^a^		13 902 (40)	7716 (52)	<.001	7975 (39)	3977 (53)	<.001	3685 (46)	1842 (56)	<.001	2242 (37)	1897 (46)	<.001	
Atrial fibrillation, *n* (%)^a^		15 278 (44)	7939 (53)	<.001	8413 (41)	3602 (48)	<.001	3522 (45)	1896 (57)	<.001	3343 (56)	2441 (60)	<.001	
Malignant cancer within 3 years, *n* (%)^a^		3742 (11)	2119 (14)	<.001	2007 (10)	1017 (14)	<.001	956 (12)	468 (14)	.004	779 (13)	634 (16)	<.001	
Valvular disease, *n* (%)^a^		5058 (15)	2986 (20)	<.001	2332 (11)	1230 (17)	<.001	1268 (16)	705 (21)	<.001	1458 (24)	1051 (26)	.089	
COPD, *n* (%)^a^		3396 (10)	1727 (12)	<.001	1834 (9)	773 (10)	<.001	732 (9)	376 (11)	<.001	830 (14)	578 (14)	.77	
Liver disease, *n* (%)^a^		685 (2)	199 (2)	<.001	424 (2)	84 (1)	<.001	121 (2)	46 (2)	.77	140 (3)	69 (2)	.033	
Musculoskeletal/connective tissue disease within 3 years, *n* (%)^a^		9131 (26)	4909 (33)	<.001	4747 (23)	2208 (29)	<.001	2281 (29)	1141 (34)	<.001	2103 (35)	1560 (38)	<.001	
Dementia, *n* (%)		333 (1)	289 (2)	<.001	160 (1)	141 (2)	<.001	62 (1)	62 (2)	<.001	111 (2)	86 (2)	.39	
Depression, *n* (%)		1225 (4)	520 (3)	.74	742 (4)	258 (3)	.53	246 (3)	104 (3)	.92	237 (4)	158 (4)	.77	
CCI, *n* (%)	**0–1**	14 360 (42)	3489 (23)	<.001	9059 (44)	1809 (24)	<.001	3162 (40)	771 (23)	<.001	2139 (36)	909 (21)	<.001	
	**2–3**	13 418 (39)	5630 (38)		7894 (38)	2941 (38)		3182 (40)	1231 (37)		2342 (39)	1458 (35)		
	**4–7**	5607 (16)	4813 (23)		3096 (15)	2324 (31)		1282 (16)	1082 (33)		1229 (21)	1407 (35)		
	**≥8**	1151 (3)	981 (7)		603 (3)	438 (6)		295 (4)	224 (7)		253 (4)	319 (8)		
**Medications**														
RASi/ARNI, *n* (%)		31 847 (92)	12 703 (85)	<.001	19 850 (96)	6799 (91)	<.001	7309 (92)	2816 (85)	<.001	4688 (79)	3088 (75)	<.001	
MRA, *n* (%)		12 383 (36)	5369 (36)	.82	8590 (42)	2930 (39)	<.001	1908 (24)	1000 (30)	<.001	1885 (32)	1439 (35)	<.001	
Digoxin, *n* (%)		4819 (14)	1980 (13)	.011	2961 (14)	982 (13)	<.001	905 (11)	433 (13)	.013	953 (16)	565 (14)	.003	
Diuretic, *n* (%)		23 162 (67)	13 039 (87)	<.001	14 225 (69)	6525 (87)	<.001	4363 (55)	2813 (85)	<.001	4574 (77)	3701 (90)	<.001	
Nitrate, *n* (%)		8602 (25)	4612 (31)	<.001	5004 (24)	2438 (32)	<.001	2340 (29)	1068 (32)	.004	1258 (21)	1106 (27)	<.001	
Platelet inhibitor, *n* (%)		16 008 (46)	7842 (53)	<.001	9690 (47)	4188 (56)	<.001	3912 (49)	1736 (52)	.003	2406 (40)	1918 (47)	<.001	
Statin, *n* (%)		16 689 (48)	7816 (52)	<.001	9880 (48)	4040 (54)	<.001	4191 (53)	1792 (55)	.22	2618 (44)	1984 (49)	<.001	
Oral anticoagulant, *n* (%)		15 699 (45)	77 101 (48)	<.001	9248 (45)	3415 (45)	.31	3445 (43)	1671 (51)	<.001	3006 (50)	2015 (49)	.24	
Beta-blockers, *n* (%)		31 070 (90)	13 427 (90)	.81	19 293 (93)	6941 (92)	.003	6887 (87)	2975 (90)	<.001	4890 (82)	3511 (86)	<.001	
Calcium channel blockers, *n* (%)		6715 (19)	4914 (33)	<.001	3185 (15)	1930 (26)	<.001	1634 (21)	1191 (36)	<.001	1896 (32)	1793 (44)	<.001	
SGLT2i, *n* (%)		947 (3)	274 (2)	<.001	708 (3)	182 (2)	<.001	168 (2)	49 (2)	.025	71 (1)	43 (1)	.51	
**Socio-economic characteristics**														
Education, *n* (%)^a^	**Compulsory school**	12 810 (37)	7381 (49)	<.001	7407 (36)	3601 (47)	<.001	2869 (36)	1634 (49)	<.001	2534 (42)	2146 (52)	<.001	
	**Secondary school**	14 957 (43)	5339 (36)		9247 (45)	2775 (37)		3395 (43)	1182 (36)		2315 (39)	1382 (34)		
	**University**	6769 (20)	2193 (15)		3998 (19)	1136 (15)		1657 (21)	492 (15)		1114 (19)	565 (14)		
Disposable income, Above median within year, *n* (%)^a^		17 947 (52)	5172 (35)	<.001	111 215 (54)	2776 (37)	<.001	4235 (53)	1162 (35)	<.001	2497 (42)	1234 (31)	<.001	
Family type, Living alone, *n* (%)^a^		15 359 (45)	7647 (51)	<.001	9113 (44)	3701 (49)	<.001	3293 (42)	1646 (50)	<.001	2953 (50)	2300 (56)	<.001	
**Clinical characteristics**														
Location, In-patient, *n* (%)		13 513 (39)	7773 (52)	<.001	7684 (37)	3539 (47)	<.001	2625 (33)	1637 (49)	<.001	3204 (52)	2597 (63)	<.001	
Follow-up referral, *n* (%)	**Hospital**	26 950 (81)	9275 (66)	<.001	17 629 (88)	5420 (75)	<.001	5967 (78)	1953 (63)	<.001	3354 (59)	1902 (51)	<.001	4
	**Primary care**	5677 (17)	4394 (31)		2058 (10)	1570 (22)		1535 (20)	1064 (34)		2084 (37)	1760 (46)		
	**Other**	771 (2)	416 (3)		391 (2)	190 (3)		172 (2)	90 (3)		208 (4)	136 (3)		
NYHA class, *n* (%)	**I-II**	18 038 (71)	5503 (56)	<.001	10 861 (68)	2855 (53)	<.001	4706 (79)	1352 (63)	<.001	2471 (69)	1296 (58)	<.001	29
	**III-IV**	7454 (29)	4296 (44)		5072 (32)	2551 (47)		1261 (21)	803 (38)		1121 (32)	942 (42)		
BMI (kg/m^2^) ≥30, *n* (%)		6618 (27)	2576 (25)	.003	3871 (25)	1150 (22)	<.001	1533 (27)	570 (26)	.13	1214 (31)	856 (32)	1.00	28
Systolic blood pressure, (mmHg), median [IQR]		125 [112, 140]	129 [115, 140]	<.001	121 [110, 140]	124 [110, 140]	.054	130 [117, 142]	130 [119, 145]	.011	130 [120, 146]	132 [120, 150]	.005	1
Diastolic blood pressure, (mmHg), median [IQR]		75 [68, 82]	70 [65, 80]	<.001	75 [68, 82]	70 [64, 80]	<.001	75 [70, 83]	72 [65, 80]	<.001	75 [67, 81]	70 [64, 80]	<.001	1
Mean arterial pressure >90 mmHg, *n* (%)		118 609 (55)	7459 (51)	<.001	10 826 (51)	3671 (46)	<.001	4759 (59)	1878 (54)	<.001	3502 (60)	2253 (56)	<.001	1
Heart rate (beats/min) > 70, *n* (%)		18 358 (54)	7965 (55)	.51	11 475 (56)	4167 (56)	.97	3835 (49)	11 714 (53)	<.001	3048 (52)	2084 (52)	.68	2
Potassium (mmol/L), *n* (%)	**Normakalemia**	28 687 (95)	11 304 (90)	<.001	17 311 (95)	5728 (90)	<.001	6717 (95)	2558 (90)	<.001	4659 (92)	3018 (89)	<.001	14
	**Hypokalemia**	1202 (4)	658 (5)		594 (3)	285 (4)		268 (4)	147 (5)		340 (7)	226 (7)		
	**Hyperkalaemia**	395 (1)	574 (5)		275 (2)	323 (5)		74 (1)	119 (4)		46 (1)	132 (4)		
Haemoglobin, (g/L), median [IQR]		138 [126, 149]	128 [116, 140]	<.001	140 [128, 150]	131 [118, 143]	<.001	137 [125, 148]	126 [114, 139]	<.001	131 [119, 142]	123 [112, 135]	<.001	39
NT-proBNP (pg/ml), Above median, *n* (%)		9108 (43)	5828 (67)	<.001	6331 (49)	3245 (73)	<.001	1533 (32)	1211 (63)	<.001	1244 (34)	1372 (57)	<.001	39

^a^Variables included as covariates in the multivariable multinomial regression model.

Hypokalaemia, normokalaemia, and hyperkalaemia were defined as serum potassium levels of <3.5, 3.5–5.0, and ≥5.0 mEq/l, respectively.

Missingness refers to the overall HF cohort with available eGFR.

Abbreviations: HF, heart failure; eGFR, estimated glomerular filtration rate; HFrEF, heart failure with reduced ejection fraction; HFmrEF, heart failure with mildly reduced ejection fraction; HFpEF, heart failure with preserved ejection fraction; IQR, interquartile range; TIA, transient ischaemic attack; COPD, chronic obstructive pulmonary disease; CCI, Charlson comorbidity index; RASi, renin-angiotensin system inhibitor; ARNI, angiotensin receptor neprilysin inhibitor; MRA, mineralocorticoid receptor antagonists; SGLT2i, sodium/glucose cotransporter-2 inhibitors; NT-proBNP, *N*-terminal pro-B-type natriuretic peptide; NYHA, New York Heart Association, BMI, body mass index.

The significance level was set at 5%, with all *P*-values calculated as two-sided. Data management and statistical analyses were conducted using Stata version 17.0 (StataCorp, College Station, Texas) and R v.4.3.1 (R Foundation for Statistical Computing, Vienna, Austria).

### Ethics

This study was approved by the Swedish Ethical Review Authority and complies with the Helsinki Declaration.

## Results

After applying the selection criteria, 103 696 patients (51 848 with and 51 848 without HF) were included in the analysis where CKD was defined according to ICD-10 diagnoses, of whom 63% were males, and the median age was 74 years (Q1–Q3: 64–81). The proportion of CKD defined according to ICD-10 codes was 2% in patients without HF and 6% in those with HF (*P*-value <.001). Among patients with HF, 57% had HFrEF, 23% HFmrEF, and 20% HFpEF, and the respective proportions of CKD in each group were 5%, 5%, and 8% (*Table 1*).

In the analyses investigating the cohort of patients with HF and available eGFR measurements, 49 449 individuals were considered. Demographics and proportions of EF phenotypes were consistent with the ICD-10–defined cohort. The prevalence of CKD based on eGFR <60 ml/min/1.73m^2^ was 30% in HF overall, 27% in HFrEF, 29% in HFmrEF, and 41% in HFpEF (*P*-value <.001, *Table 3*). The distribution of KDIGO stages was as follows: 60% G2 (eGFR 60–89), 22% G3a (45–59), 13% G3b (30–44), 4% G4 (15–29), and 1% G5 (<15) (Supplementary Table S2).

### Patient characteristics (*Table 1–3*)

Baseline characteristics of HF vs non-HF patients and of HF patients according to EF are reported in *Table 1*. Most patient characteristics were statistically significantly different between patients with vs without CKD in both HF and non-HF groups due to the large sample size. In patients with new-onset HF, those with CKD were older, had a higher burden of cardiometabolic comorbidities, including diabetes, hypertension, and ischaemic heart disease, and had lower education and income. As regards medications, patients with vs without CKD were more frequently prescribed drugs for congestion and comorbidity management, but were less likely to receive renin–angiotensin system inhibitors (RASi)/angiotensin receptor neprilysin inhibitors (ARNI), mineralocorticoid receptor antagonists (MRA), digoxin, and oral anticoagulants. These differences were generally consistent across the EF spectrum, except for education level within the individual phenotypes, age in HFpEF, and oral anticoagulant use in HFmrEF, which did not differ by CKD status (*Table 2*).

In the group without HF, differences between individuals with and without CKD were overall consistent with those observed among patients with new-onset HF: CKD identified a population with higher comorbidity burden and greater medication use, except for MRA, digoxin, and sodium-glucose transport protein 2 inhibitors (SGLT2i), whose use was consistent regardless of CKD.

Compared with ICD-10 codes-defined CKD, those defined according to an eGFR <60 ml/min/1.73 m^2^ were slightly older, but overall represented a healthier HF population, with fewer comorbidities and more frequent use of RASi/ARNI or MRA.

Patient characteristics according to the CKD status defined as eGFR < vs ≥60 ml/min/1.73m^2^ are reported in *Table 3* and were overall consistent with what was observed in the ICD-10 cohort. Across KDIGO categories, patients with G5 CKD, as compared with G2–G4, had a more clinically and socioeconomically vulnerable profile, with higher comorbidity burden, lower education and income, greater use of medications for congestion and comorbidity management, and lower use of RASi/ARNI, MRA, digoxin, and oral anticoagulants (Supplementary Tables S2 and S3).

### Association between CKD and HF

#### CKD defined according to ICD-10 codes

**Figure 1:**
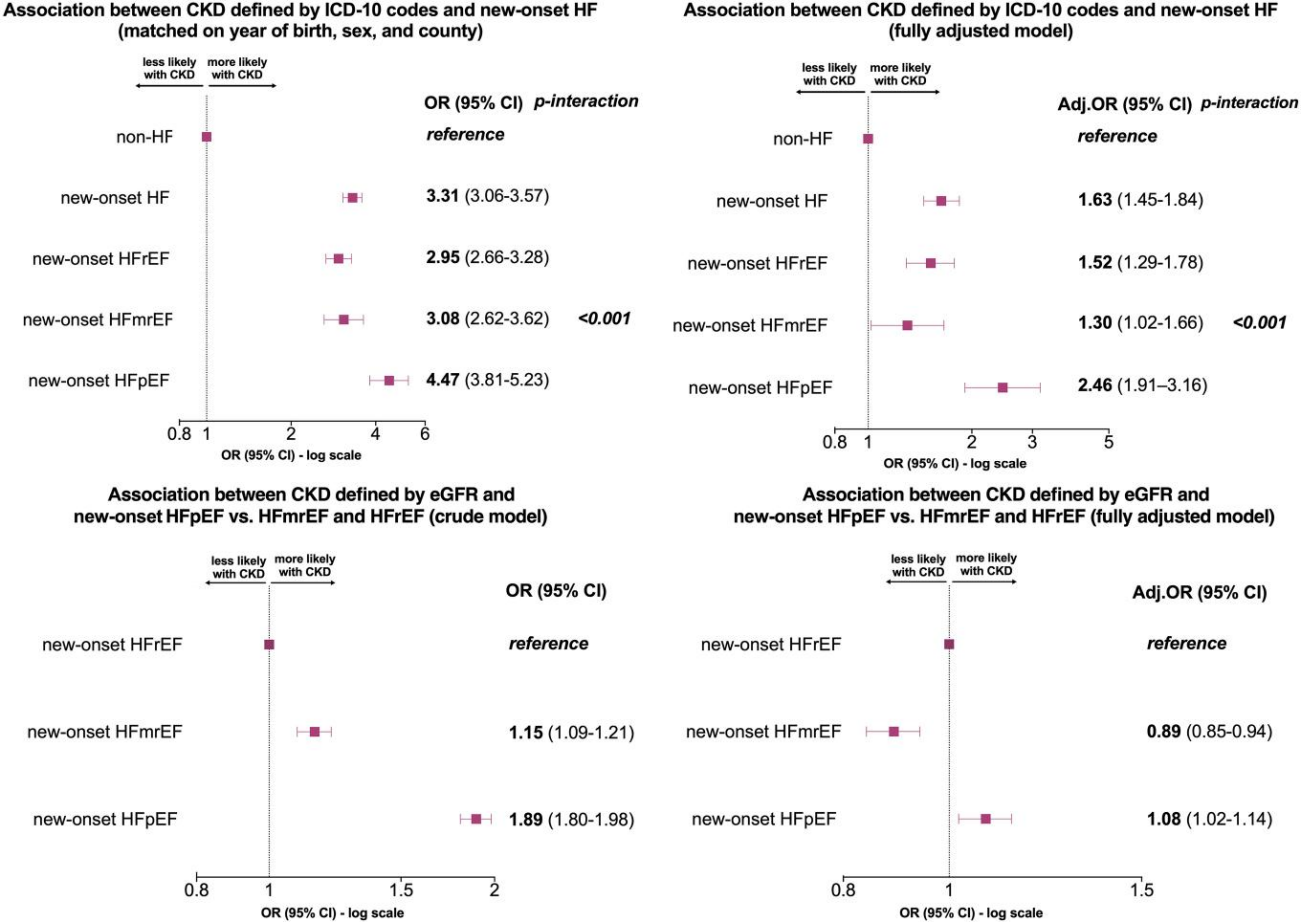
Associations between CKD defined by ICD-10 codes and by eGFR and HF according to EF. Odds ratio with respect to having CKD. Dependent variable: HF or HF across the EF spectrum; independent variable: CKD defined by ICD-10/procedural codes or CKD defined as eGFR <60 ml/min/1.73 m^2^. Reference category: non-HF for ICD −10 analysis; HFrEF for eGFR <60 ml/min/1.73 m^2^. Abbreviations: HF, heart failure; CKD, chronic kidney disease; HFrEF, heart failure with reduced ejection fraction; HFmrEF, heart failure with mildly reduced ejection fraction; HFpEF, heart failure with preserved ejection fraction; OR, odds ratio; CI, confidence intervals; *P*, *P*-value, eGFR, estimated glomerular filtration rate

CKD defined according to ICD-10 codes was associated with a crude ∼three-fold (OR 3.31, 95% CI 3.06–3.57) and adjusted ∼two-fold (OR 1.63, 95% CI 1.45–1.84) higher odds of new-onset HF as compared with no CKD. Notably, CKD was more strongly linked to new-onset HFpEF (OR 2.46, 95% CI 1.91–3.16 for HFpEF vs. non-HF) vs HFrEF (OR 1.52, 95% CI 1.29–1.78 for HFrEF vs. non-HF) and HFmrEF (OR 1.30, 95% CI 1.02–1.66 for HFmrEF vs. non-HF) (*P*-value for interaction <.001) (*Figure 1*).

#### CKD defined as eGFR <60 ml/min/1.73m^2^

**Figure 2:**
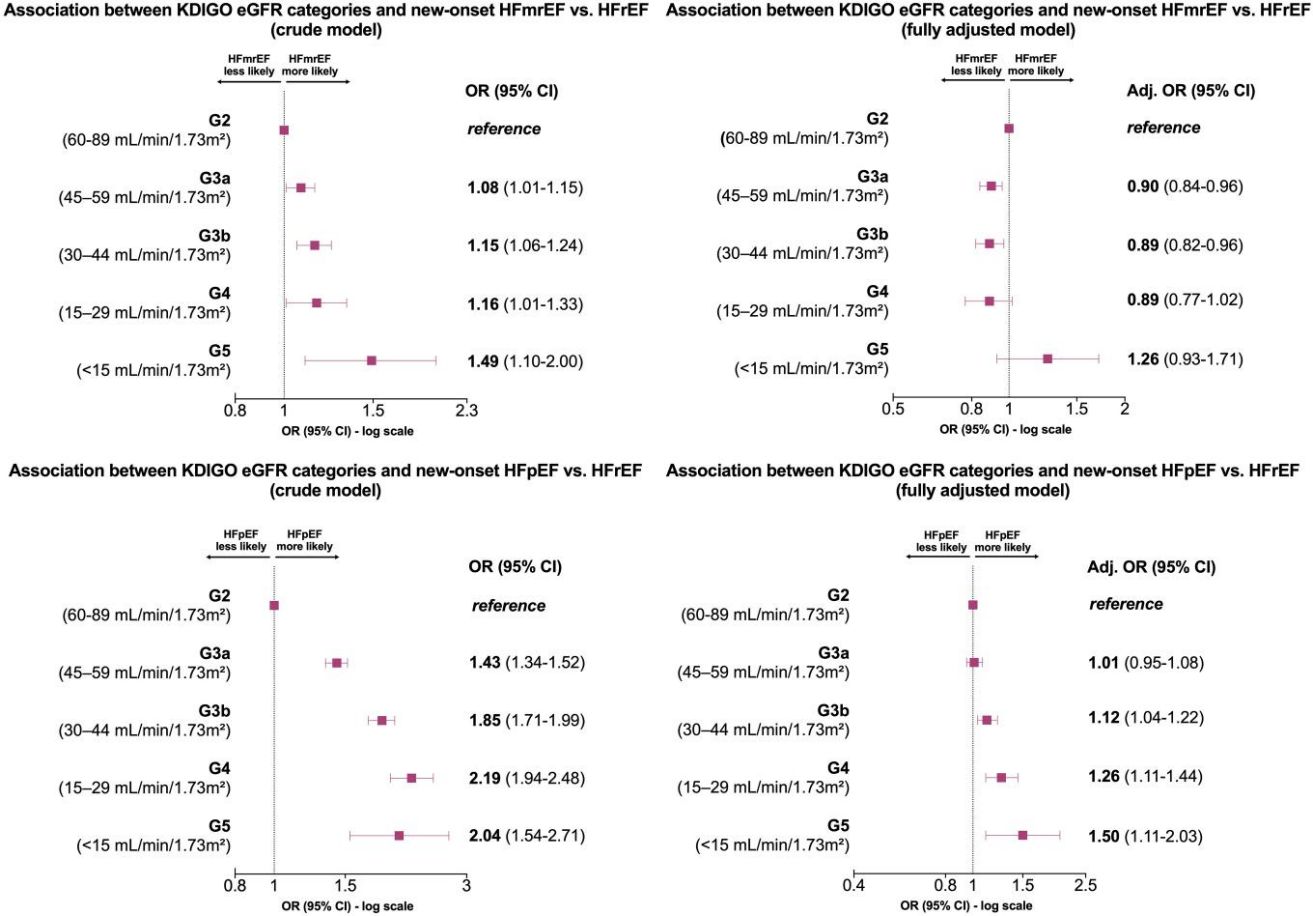
Associations between CKD severity and HF according to EF. Odds ratios for new-onset HFmrEF and HFpEF with respect to having CKD stratified by KDIGO categories, dependent variable: HF across the EF spectrum, independent variable: KDIGO eGFR categories: G3a (45–59 ml/min/1.73 m^2^), G3b (30–44 ml/min/1.73 m^2^), G4 (15–29 ml/min/1.73 m^2^), and G5 (<15 ml/min/1.73 m^2^). Within each model, the reference eGFR category was G2 (60–89 ml/min/1.73 m^2^). Reference category for EF: HFrEF. Abbreviations: eGFR, estimated glomerular filtration rate; HFmrEF, heart failure with mildly reduced EF; HFpEF, heart failure with preserved EF; OR, odds ratio; CI, confidence interval

CKD defined as eGFR <60 ml/min/1.73m^2^ was associated with a crude 89% higher odds of new-onset HFpEF and 15% higher odds of HFmrEF as compared with HFrEF (*Figure 1*). After adjustments, CKD was associated with 8% higher odds of new-onset HFpEF (OR 1.08, [95% CI 1.02–1.14]) and 11% lower odds of new-onset HFmrEF (OR 0.89, [95% CI 0.85–0.94]). When stratified by KDIGO categories, using G2 as reference, G3a–G5 stages were associated with a crude 1.4–2.2-fold higher odds of new-onset HFpEF vs. HFrEF, and with 1.1–1.5-fold higher odds of new-onset HFmrEF vs HFrEF (*Figure 2*). After adjustments, as compared with G2, G3a had similar odds of HFpEF and HFrEF (OR 1.01, [95% CI 0.95–1.08]; whereas G3b (OR 1.12, [95% CI 1.04–1.22]), G4 (OR 1.26, [95% CI 1.11–1.44]) and G5 (OR 1.50, [95% CI 1.11–2.03]) had greater association with HFpEF vs HFrEF. For HFmrEF, G3a (OR 0.90, [95% CI 0.84–0.96]) and G3b (OR 0.89, [95% CI 0.82–0.96]) had greater association with HFrEF than HFmrEF, whereas G4 (OR 0.89, [95% CI 0.77–1.02]) and G5 (OR 1.26, [95% CI 0.93–1.71]) were similarly associated with HFmrEF and HFrEF.

#### Patient characteristics associated with new-onset HF in patients with vs. without CKD (*Figure 3*, Supplementary Table S4)

History of atrial fibrillation, valvular disease, ischaemic heart disease, hypertension, chronic obstructive pulmonary disease (COPD), liver disease, diabetes, peripheral artery disease, musculoskeletal/connective tissue disease, malignant cancer, and the absence of prior stroke or transient ischaemic attack (TIA) were independently associated with a higher odds of new-onset HF.

**Figure 3:**
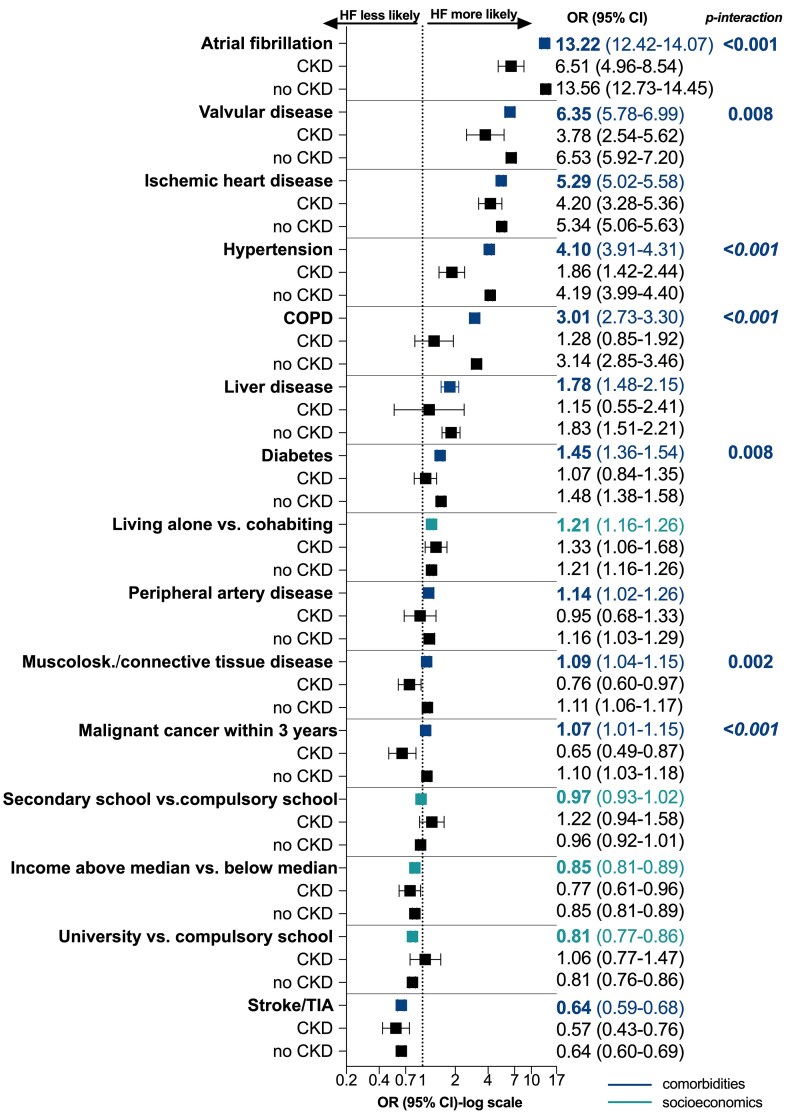
Patient characteristics associated with new-onset HF according to CKD status. All covariates were tested for interaction with CKD; only significant results are displayed in the graph. The full results of the interaction analyses across HF phenotypes are reported in Supplementary Table S4. Abbreviations: HF, heart failure; CKD, chronic kidney disease; OR, odds ratio; CI, confidence intervals; *P*, *P*-value; COPD, chronic obstructive pulmonary disease; TIA, transient ischaemic attack, muscolosk./connective tissue disease, musculoskeletal or connective tissue disease

Socioeconomic factors, including living alone and lower income and higher education, were also significantly associated with higher odds of new-onset HF.

Factors independently linked with new-onset HF were largely consistent regardless of the prevalent CKD, except for musculoskeletal/connective tissue disease and malignant cancer, which showed lower odds of HF in patients with CKD.

#### Patient characteristics associated with new-onset HFpEF, HFmrEF vs HFrEF according to CKD status (Supplementary Figures S2 and S3, Table S4)

As compared with HFrEF, patients with ischaemic heart disease were less likely to develop HFpEF, while liver disease, peripheral artery disease and higher education were not associated with different odds of HFpEF vs. HFrEF. Similarly, lower income, living alone, history of stroke/TIA and COPD were not associated with a similar odds of HFmrEF vs. HFrEF.

Most patient characteristics associated with new-onset HF were similar among HFpEF, HFmrEF, and HFrEF in patients with vs without CKD, with key exceptions being diabetes, which was associated with a higher odds of new-onset HFmrEF vs HFrEF only in patients with CKD.

## Discussion

In a large, nationwide contemporary cohort, we identified the following key findings: (i) CKD was independently associated with new-onset HF, with a crude ∼three-fold and adjusted two-fold higher odds as compared with patients without CKD; (ii) CKD was associated with new-onset HF across the EF spectrum, but the magnitude of the association was greatest with HFpEF, except that for G3a stage, which was similarly associated with HFrEF and HFpEF; (iii) key patients characteristics associated with new-onset HF across the EF spectrum were largely similar across CKD status.

### Association between CKD and new-onset HF

In adjusted analysis, patients with prevalent CKD had double the odds of new-onset HF vs those without CKD. Similar findings were reported in the Atherosclerosis Risk in Communities (ARIC) and the Physicians’ Health Study, where a two-fold higher HF risk was observed in patients with moderate to severe CKD (eGFR <60 ml/min/1.73 m^2^) over 13.2 and 10 years of follow-up, respectively.^3,10^ Conversely, the Framingham Heart Study (FHS) found no association between CKD, defined as serum creatinine 1.4–3.0 mg/dl, and risk of HF over 15 years, possibly due to a lower-risk population, limited power, and the use of serum creatinine rather than eGFR, which may have underestimated the prevalence of CKD and its association with HF.^11^

Our study expands the current knowledge in the field by providing new insights from a large, unselected population, where the availability of granular data allowed detailed analyses. First, our exposure was a clinical diagnosis of CKD rather than isolated eGFR or creatinine measurements, which may reduce misclassification, increase specificity for true CKD cases, and avoid reliance on single laboratory values that may not reflect persistent kidney dysfunction.^10,11^ Second, while most prior studies defined HF based on the occurrence of related hospitalizations or death, we also considered outpatient diagnoses of HF, which reduced selection bias towards more severe presentations of HF.^3,15^ Third, we leveraged multiple data sources, enhancing accuracy and coverage.

Several possible factors may explain the association between CKD and new-onset HF. CKD and HF share numerous risk factors, such as history of hypertension, atrial fibrillation, ischaemic heart disease, COPD, musculoskeletal/connective tissue disease, malignancy, and low socioeconomic status.^2,20–23^ However, the remarkably strong association we observed between CKD and new-onset HF persisted even after adjusting for risk factors and other patient characteristics, which could have confounded the analysis: this finding contrasts with prior reports.^24^ Although our study cannot directly assess underlying mechanisms, prior evidence suggests that volume overload, neurohormonal activation, inflammation, and endothelial dysfunction, all key pathophysiological factors that do characterize CKD and are also related to disease severity, might indeed be involved in the pathogenesis of HF and therefore mediate the link between CKD and the risk of HF onset.^25^ Our data might contribute to highlight the importance of treating CKD with pharmacological agents shown to prevent HF in patients with CKD, such as SGLT2i, non-steroidal MRA and glucagon-like peptide-1 receptor agonists.^9^

### Association between CKD and HF across the EF spectrum

In our analysis, CKD had a greater association with new-onset HFpEF vs HFmrEF or HFrEF, regardless of the definition applied. Nevertheless, the association between eGFR-defined CKD and new-onset HFpEF was modest in magnitude. When we stratified by CKD severity according to KDIGO categories, moderate to advanced CKD (G3b–G5) was more strongly associated with new-onset HFpEF, whereas mild CKD (G3a) was similarly associated with HFrEF and HFpEF.

Our results support the notion that different EF phenotypes may arise from distinct pathophysiological mechanisms. CKD may amplify key drivers for HFpEF, including ageing, inflammation, fibrosis, microvascular dysfunction, and haemodynamic stress, which are hallmarks of both conditions.^26^ By contrast, CKD may contribute to HFrEF through fluid overload, more dominant neurohormonal activation, and myocardial remodelling.^26^ In moderate to advanced CKD, a combination of microvascular dysfunction, arterial stiffening, systemic inflammation, neurohormonal activation, and accumulation of uraemic toxins promotes myocardial fibrosis, concentric remodelling, and diastolic stiffness, thereby increasing the likelihood of developing HFpEF.^27^ In contrast, the mechanisms linking mild CKD to HF remain less clearly defined.^15,26^

One hypothesis is that in early stages of CKD, the degree of renal impairment may not be sufficient to trigger the distinct haemodynamic and microvascular changes typical of HFpEF, and modest elevations in preload and left ventricular filling pressure may contribute to both HFpEF and HFrEF risk.^28^

Prior reports only partly align with our findings, likely due to heterogeneous CKD definitions (e.g. eGFR, albuminuria, cystatin C) and limited stratification by CKD severity.^2,15–17^ In the FHS study, CKD, defined as an eGFR <60 ml/min/1.73m^2^, was associated with a slightly higher risk of HF overall, without differences between incident HFpEF or HFrEF after adjustment, whereas microalbuminuria was specifically linked with the risk of HFrEF over 15 ± 4 years of follow-up.^16^ In the Women’s Health Initiative (WHI), which followed over 23 000 post-menopausal women for nearly 19 years, there was a stepwise increase in risk for incident HF with declining renal function, with similar risks for HFpEF and HFrEF at eGFR 45–59 ml/min/1.73 m^2^, but a stronger association for HFpEF at eGFR <45 ml/min/1.73 m^2^.^29^

Similarly, the PREVEND study found that CKD, defined by urinary albumin excretion and cystatin C, was specifically linked with incident HFpEF over 11.5 years of follow-up.^2^ Discrepancies may stem from differences in cohort selection (healthier participants in FHS vs higher-risk groups in PREVEND and WHI), renal biomarkers (microalbuminuria, considered a potent activator of the renin–angiotensin–aldosterone system and closely linked with HFrEF vs UAE and cystatin C, more sensitive markers of early renal impairment associated with HFpEF), CKD definitions, and statistical power limitations.^2,15,16,29^

Few studies have specifically addressed whether the association between CKD and EF phenotypes differs according to CKD severity.

In the CRIC study, which included participants with eGFR ranging from 20 to 70 ml/min/1.73 m^2^, incident rates of HFpEF were consistently higher than those of HFrEF across all eGFR categories (<45, 30–44, and <30 ml/min/1.73 m^2^), with risk increasing progressively as renal function declined.^17^ In a cross-sectional cohort of 12 621 hospitalized patients with new-onset HF across the full eGFR spectrum, the adjusted rates of HFpEF increased from 54% among patients with eGFR ≥60 ml/min/1.73 m^2^ to 70% among those with eGFR <15 ml/min/1.73 m^2^ (*P* for trend = .02).^18^

Similarly, in a longitudinal substudy of the CRIC cohort examining cardiac remodelling during the transition from advanced CKD (eGFR <20 ml/min/1.73 m^2^) to end-stage renal disease, defined as the need for chronic dialysis, the proportion of participants with HFpEF increased from 33% to 46% over 2 years of follow-up, supporting a continuum in which progressive renal dysfunction predisposes to incident HFpEF.^30^

The inclusion of HFmrEF as a separate category further sets our study apart from previous reports, as this EF phenotype has typically been grouped with either HFrEF or HFpEF or completely neglected.^2,4,15,16^ In our analysis, CKD had similar associations with HFmrEF and HFrEF, except for G3a–G3b, which was more strongly linked to HFrEF. This reinforces the concept that HFmrEF is more similar for many aspects to HFrEF vs HFpEF, including risk factors and prognosticators, though its pathophysiology remains less defined.^13^

### Patient characteristics associated with new-onset HF in patients with vs without CKD

In the overall population, patients with a history of atrial fibrillation, valvular disease, ischaemic heart disease, hypertension, COPD, liver disease, diabetes, peripheral artery disease, musculoskeletal/connective tissue disease, malignant cancer within three years, and the absence of prior stroke or TIA were associated with higher odds of new-onset HF, consistent with prior investigation.^2,19^

Patient characteristics linked with new-onset HF did not substantially differ according to CKD status, except for musculoskeletal/connective-tissue disease and cancer, which were associated with lower odds of HF only in patients with CKD. This finding might be at least partially explained by competing risks, as CKD independently predicts mortality in these groups, and many patients may die before HF can develop or be documented.^31,32^

Patient characteristics independently associated with new-onset HF were largely consistent across HFpEF, HFmrEF or HFrEF, with few significant exceptions. In HFpEF, patients were less likely to have ischaemic heart disease compared with those with HFrEF, suggesting that HFpEF is more closely associated with microvascular dysfunction and inflammation rather than macrovascular disease.^26^ Independent characteristics linked with new-onset HF across the EF spectrum were generally similar regardless of prevalent CKD. The key exception was diabetes, which showed greater associations with HFmrEF compared with HFrEF in patients with CKD. One potential explanation is that diabetes largely promotes microvascular dysfunction and myocardial fibrosis. When CKD is present, these pathophysiological processes may be amplified by systemic low-grade inflammation, which can lead to diastolic dysfunction and myocardial stiffness, favouring the development of HFmrEF over HFrEF.^33,34^ In addition, among patients with CKD, those with HFmrEF received less intensive pharmacological management than those with HFrEF, particularly regarding the use of RASi/ARNI, MRA, and platelet inhibitors, which may have further amplified the adverse myocardial effects of diabetes in this cohort.

### Limitations

As a retrospective, registry-based, matched case-control study, this investigation cannot infer causality due to potential residual confounding despite adjustments and matching. A proportion of CKD cases might be misclassified as no CKD since CKD was defined according to ICD-10 code diagnoses from secondary/tertiary care, while CKD in Sweden is often diagnosed and managed in primary care until referral to specialty care is required.^35^ While this might have diluted the association between CKD and new-onset HF, the inclusion of patients with likely more severe CKD followed up in secondary/tertiary care may have led to an overestimation of the magnitude of the association. However, our analyses were confirmed when CKD was defined according to eGFR, although this approach was likely cross-sectional, as eGFR could have been measured close to the HF diagnosis. Therefore, eGFR-defined CKD may partly reflect acute haemodynamic changes or kidney injury related to HF rather than pre-existing CKD. Although patients with acute kidney disease codes during the year before the index were excluded, residual misclassification cannot be ruled out, potentially limiting the temporal interpretation of the eGFR-based analyses. Our study period did not consider the introduction of SGLT2i for the treatment of CKD, which has been shown to lower the risk of HF.^9,36,37^ Markers of kidney damage, including albuminuria, were not available and this may have further resulted in underestimation of CKD and potential misclassification of G2 (used as the reference category in KDIGO-stratified analyses). The external validity of these findings may be affected by differences in healthcare systems, clinical management, and population characteristics outside Sweden.

## Conclusions

CKD was significantly associated with new-onset HF, especially HFpEF over other EF phenotypes in moderate to advanced CKD, and HFpEF and HFrEF in mild CKD. Patient characteristics linked with new-onset HF across the EF spectrum did not substantially differ according to CKD status, which further supports the independent relationship between CKD and HF. The greater association observed with HFpEF, even after adjustment for risk factors and patient characteristics, is consistent with the hypothesis that CKD may be more than a coexisting condition but rather a risk factor for HFpEF. Our findings have important implications for the management of patients with CKD. The prevention of HF in this high-risk population should be considered a public health priority, especially as several new therapies for CKD, including SGLT2i, non-steroidal MRA, and glucagon-like peptide-1 receptor agonists, have demonstrated efficacy in preventing HF onset and mitigating its overall burden.^36–39^

## Supplementary Material

xvag196_Supplementary_Data

## Declarations

### Data availability

The data underlying this article will be shared on reasonable request to the corresponding author.

### Pre-registered Clinical Trial Number

None supplied.
