# Supplementary material for: Association between chronic kidney disease and heart failure across the ejection fraction spectrum: a retrospective case-control study from the Swedish Heart Failure Registry
Source: ESC Heart Fail. 2026 Jul 19;13(4):xvag196. doi: 10.1093/eschf/xvag196 (PMC13395074; doi:10.1093/eschf/xvag196)
Supplement: xvag196_Supplementary_Data [file xvag196_supplementary_data.docx]

**Supplementary Materials**

**Association Between Chronic Kidney Disease and Heart Failure Across the Ejection Fraction Spectrum: A Retrospective Case-Control Study from the Swedish Heart Failure Registry**

Valeria Valente^1^, Lina Benson^1,2^, Carin Corovic Cabrera^1^, Raffaele Scorza^1^, Felix Lindberg^1^, Ida Haugen Löfman^2^, Michael Melin^1^, Lars H. Lund^2^, Giulia Ferrannini^2,3^, Gianluigi Savarese^1^

^1^ Department of Clinical Science and Education, Södersjukhuset; Karolinska Institutet, Stockholm, Sweden

^2^ Division of Cardiology, Department of Medicine; Karolinska Institutet, Stockholm, Sweden

^3^ Internal Medicine Unit, Södertälje Hospital, Södertälje, Sweden

***Data sources***

SwedeHF was established in 2000, and is an ongoing nationwide quality registry aiming at improving the management and outcome of patients with HF in Sweden.^16^ It collects ~100 variables including demographics, comorbidities, key clinical and organizational variables and laboratory measurements, and treatments. Until April 2017, patients were included based on an HF diagnosis defined according to the treating physician. Since April 2017, the HF diagnosis has been defined according to ICD-10 codes (I50.0, I50.1, I50.9, I42.0, I42.6, I42.7, I25.5, I11.0, I13.0, and I13.2).^16^ In 2021 (last year of patient enrolment for the current study), coverage was 32% of the prevalent HF population in Sweden.^17^ Individual patient consent is not required for registration in SwedeHF, however, patients are informed of their inclusion and can decide to opt out.

For the purpose of the current analysis, SwedeHF was linked with other national registers by using the unique personal identification numbers that all Swedish residents have. The National Patient Register, which serves as a national administrative record of hospital admissions and outpatient visits but does not include primary care, provided additional comorbidities and data on CKD, i.e. the study exposure, as ICD-10 diagnoses. The Swedish Prescribed Drug Register provided data on pharmacological treatment, with a patient considered to be on a specific treatment whether at least one dispensation was recorded between five months prior and 14 days after the index date. From the Cause of Death Register we obtained information on vital status and cause of death. The Longitudinal Integrated Database for Health Insurance and Labor Market Studies (LISA) provided socio-economic data, including education, income, and family type. Statistics Sweden provided the control population without HF.

**Table S1a – Definition of CKD according to ICD-10 or procedural codes**

| **ICD 10/**  **procedure codes** | **Definitions** | **Source** | **Period** |
| --- | --- | --- | --- |
| N18 | Chronic renal failure | SwedeHF^4^, Friberg (2018)^5^, Centers for Medicare and Medicaid Services^6^, Savarese (2023)- Evolution HF^7^, Sundström (2021)-CELOSIA^8^, Ludvigsson (2021)^9^ | 5 years before- prior to index date  5 years before- prior to index date  5 years before- prior to index date  5 years before- prior to index date |
| N18.1-5 | Chronic renal failure stage 1-5 | SwedeHF^4^, Friberg (2018)^5^, Centers for Medicare and Medicaid Services^6^, Savarese (2023)- Evolution HF^7^, Sandhu (2021)^10^ |  |
| N19 | Renal failure, unspecified as acute or chronic | Ludvigsson (2021)^9^ ,Friberg (2018)^5^, Ludvigsson (2021)^9^, Sandhu (2021)^10^ |  |
| Z491 | Extracorporeal dialys | SwedeHF^4^, Geng (2022)-UK biobank^11^, Sundström (2021)-CELOSIA^8^ |  |
| Z492 | Other dialysis | SwedeHF^4^, Geng (2022)-UK biobank^11^, Savarese (2023)- Evolution HF^7^, Sundström (2021)-CELOSIA^8^ |  |
| DR014 | Hemodiafiltration (HDF) | SwedeHF^4^, Sundström (2021)-CELOSIA^8^ |  |
| DR016 | Hemodialys, Chronic | SwedeHF^4^, Friberg (2018)^5^, Sundström (2021)-CELOSIA^8^ |  |
| DR020 | Continuous arteriovenous or venovenous hemofiltration or hemodiafiltration | SwedeHF^4^ |  |
| DR012 | CAPD (continuous ambulatory peritoneal dialysis) - control | SwedeHF^4^ |  |
| DR013 | CAPD - start | SwedeHF^4^, Sundström-CELOSIA (2021)^8^ |  |
| DR024 | Peritoneal dialysis, chronic | SwedeHF^4^, Friberg (2018)^5^, Sundström (2021)-CELOSIA^8^ |  |
| TJA33 | Percutaneous insertion of a peritoneal dialysis catheter | SwedeHF^4^, Sundström (2021)-CELOSIA^8^ |  |
| TJA35 | Removal of peritoneal dialysis catheter | SwedeHF^4^, Sundström (2021)-CELOSIA^8^ |  |
| Y84.1 | Renal dialysis as cause of abnormal reaction or late complication in patient unrelated to mishap at time of procedure | Geng (2022)-UK biobank^11^ |  |
| T82.4 | Mechanical complication in blood vessels of dialysis catheters | Geng (2022)-UK biobank^11^ |  |
| Z49.0 | Preparatory care for dialysis | Geng (2022)-UK biobank^11^, Sandhu (2021)^10^ |  |
| Z992 | Dialysis dependency | Friberg (2018)^5^, Geng (2022)-UK biobank^11^, Savarese (2023)- Evolution HF^7^, Ludvigsson (2021)^9^ |  |
| Z492 | Adjustment and management of vascular access device | SwedeHF^4^, Friberg (2018)^5^, Ludvigsson (2021)^9^ |  |
| DR055 | Citrate dialysis | Sundström (2021)-CELOSIA^8^ |  |
| DR056 | Heparin free dialysis | Sundström (2021)-CELOSIA^8^ |  |
| DR060 | Home dialysis control | Sundström (2021)-CELOSIA^8^ |  |
| DR061 | Home dialysis start | Sundström (2021)-CELOSIA^8^ |  |
| QF006 | Peritoneal dialysis | Friberg (2018), Sundström (2021) -CELOSIA^8^ |  |
| I12.0 | Hypertensive renal disease with renal failure | Centers for Medicare and Medicaid Services^6^, Savarese (2023)- Evolution HF^7^, Sundström (2021)-CELOSIA^8^,US Renal Data System^12^, Ludvigsson (2021)^9^, Sandhu (2021)^10^ |  |
| I13.0 | Hypertensive heart and kidney disease with heart failure | Sandhu (2021)^10^ |  |
| I13.1 | Hypertensive heart and kidney disease without heart failure | Centers for Medicare and Medicaid Services^6^, Evolution HF- Savarese (2023)^7^, US Renal Data System^12^, Ludvigsson (2021)^9^. Sandhu (2021)^10^ |  |
| I13.2 | Hypertensive heart and kidney disease with both heart failure and kidney failure (only for HF cohort) | Centers for Medicare and Medicaid Services^6^, Evolution HF- Savarese (2023)^7^, US Renal Data System^12^, Sandhu (2021)^10^ |  |
| N08.5 | Glomerular disease states in systemic connective tissue diseases | US Renal Data System^12^ |  |
| M10.3 | Gout caused by impaired kidney function | Centers for Medicare and Medicaid Services^6^, US Renal Data System^12^ |  |
| E12.2 | Malnutrition-related diabetes mellitus with kidney complications | Savarese (2023)- Evolution HF^7^, Sundström (2021)-CELOSIA^8^ |  |
| E11.2 | Diabetes mellitus type 2 - With kidney complications | Centers for Medicare and Medicaid Services^6^, Savarese (2023)- Evolution HF^7^, US Renal Data System^12^ |  |
| E13.2 | Other specified diabetes mellitus with kidney complications | Savarese (2023)- Evolution HF^7^, US Renal Data System^12^ |  |
| E102 | Diabetes mellitus type 1 with kidney complications | Sandhu (2021)^10^ |  |
| E14.2 | Unspecified diabetes mellitus with kidney complications | Savarese (2023)- Evolution HF^7^ |  |
| R94.4 | Abnormal results of kidney function studies | Centers for Medicare and Medicaid Services^6^, US Renal Data System^12^ |  |
| N02 | Recurrent and persistent hematuria (blood in the urine) - Mild glomerular abnormality | US Renal Data System^12^, Savarese (2023)- Evolution HF^7^ |  |
| N06 | Isolated proteinuria with specified morphological damage | Geng (2022)-UK biobank^11^, US Renal Data System^12^, Savarese (2023)- Evolution HF^7^ |  |
| N25 | Diseases caused by impaired function in the renal tubules | Centers for Medicare and Medicaid Services^6^, Ludvigsson (2021)^9^ |  |
| N11 | Chronic tubulo-interstitial nephritis | Geng (2022)-UK biobank^11^, US Renal Data System^12^, Savarese (2023)- Evolution HF^7^, Ludvigsson (2021)^9^ |  |
| N14 | Tubulo-interstitial and tubular kidney diseases caused by drugs and heavy metals | Geng (2022)-UK biobank^11^, Centers for Medicare and Medicaid Services^6^,US Renal Data System^12^, Savarese (2023)- Evolution HF^7^ |  |
| N15 | Other renal tubulo-interstitial diseases | Geng (2022)-UK biobank^11^, Centers for Medicare and Medicaid Services^6^, Savarese (2023)- Evolution HF^7^ |  |
| N16 | Tubulo-interstitial renal diseases in diseases classified elsewhere | Geng (2022)-UK biobank^11^, Savarese (2023)- Evolution HF^7^ |  |
| Q61. 1-3 | Polycystic kidney | Centers for Medicare and Medicaid Services^6^, US Renal Data System^12^, Ludvigsson (2021)^9^ |  |
| Q60-Q64 | Congenital malformations of the urinary organs | Centers for Medicare and Medicaid Services^6^, Geng (2022)-UK biobank^11^ |  |
| N03 | Chronic glomerulonephritis | Geng (2022)-UK biobank^11^, Centers for Medicare and Medicaid Services^6^, US Renal Data System^12^, Savarese (2023)- Evolution HF^7^, Ludvigsson (2021)^9^ |  |
| N04 | Nephrotic syndrome | Centers for Medicare and Medicaid Services^6^, US Renal Data System^12^, Savarese (2023)- Evolution HF^7^ |  |
| N06 | Isolated proteinuria with specified morphological damage | Geng (2022)-UK biobank^11^, Centers for Medicare and Medicaid Services^6^, US Renal Data System^12^, Savarese (2023)- Evolution HF^7^ |  |
| N07 | Hereditary nephropathy not elsewhere classified | Centers for Medicare and Medicaid Services^6^, US Renal Data System^12^, Savarese (2023)- Evolution HF^7^ |  |
| N08 | Glomerular disease states in diseases classified elsewhere | Geng (2022)-UK biobank^11^, Centers for Medicare and Medicaid Services^6^,US Renal Data System^12^, Savarese (2023)- Evolution HF^7^ |  |
| K76.7 | Hepatorenal syndrome | Centers for Medicare and Medicaid Services^6^, US Renal Data System^12^ |  |
| N25.0 | Renal osteodystrophy | Centers for Medicare and Medicaid Services^6^, US Renal Data System^12^ |  |

Abbreviations: ICD-10, International Classification of Diseases, Tenth Revision codes; SwedeHF, Swedish Heart Failure Registry; US, United states of America; UK, United Kingdom.

**Table S1b- Definitions of HF, prevalent comorbidities, socioeconomics, medications and clinical variables**

| **Variables** | **Definition (ICD-10 codes/ ATC)** | **Registry** | **Period** |
| --- | --- | --- | --- |
| **Outcomes** | | | |
| HF | Diagnosis in Swedish Heart Failure Registry | Swedish Heart Failure Registry |  |
| HFrEF, HFmrEF, HFpEF |  |  |  |
| **History and comorbidities** | | | |
| Alcohol | E244, E52, F10, G312, G621, G721, I426, K292, K70, K860, O354, P043, Q860, T51, Z502, Z714, Y90, Y91 | National Patient Register | 5 years before-index date |
| Diabetes | E10-4 |  |  |
| Peripheral artery disease | I70-3 |  |  |
| Hypertension | I10-5 |  |  |
| Stroke /TIA | 430-4, 438, I60-4, I69.0-4, G45 |  |  |
| Ischemic heart disease | 410-4, I20-5 |  |  |
| Atrial fibrillation | I48 |  |  |
| Malignant cancer within three years | C |  |  |
| Valvular disease | I05-8, I34-9, Q22, Q23.0-3, Q23.0-3, Q23.5-9, Z95.2-4 |  |  |
| COPD | J40-4 |  |  |
| Obstructive sleep apnea | G47.3 |  |  |
| Liver disease | B18, I85, I86.4, I98.2, K70, K71.0, K71.1, K71.3-7, K7.2-4, K76.0, K76.2-9 |  |  |
| Musculoskeletal/connective tissue disease within 3 years | M |  |  |
| Dementia | F00-4, R54 |  |  |
| Depression | F32-4 |  |  |
| **Medications** | | | |
| RASi /ARNI | C09A, C09B, C09C, C09D, C09DX04 | Swedish Prescribed Drug Register | 5 months before until 14 days after the index date |
| Beta-blocker | C07 |  |  |
| Diuretic | C03 exc DA, C03EB, C07B, C07C, C07D, C08GA, C09BA, C09DA, C09DX01 |  |  |
| Statin | C10 |  |  |
| MRA | C03DA |  |  |
| Digoxin | C01AA05 |  |  |
| Nitrate | C01DA |  |  |
| SGLT2i | A10BK, A10BD15, A10BD16, A10BD19, A10BD20, A10BD21, A10BD23, A10BD24, A10BD25 |  |  |
| Platelet inhibitor | B01AC |  |  |
| Oral anticoagulant | B01A excl C. |  |  |
| Calcium channel blockers | C08, C07FB, C09BB, C09DB, C09DX01 |  |  |
| **Socio-economic characteristics** | | | |
| Education |  | Longitudinal integrated database for health insurance and labour market studies (LISA) | Year before-index date |
| Disposable income |  |  |  |
| Family type |  |  |  |
| **Clinical characteristics** | | | |
| Location | Variable in Swedish Heart Failure Registry | Swedish Heart Failure Registry | Index date |
| Follow-up referral |  |  |  |
| NYHA class |  |  |  |
| BMI (kg/m²) >=30 |  |  |  |
| Systolic blood pressure |  |  |  |
| Diastolic blood pressure |  |  |  |
| Mean arterial pressure >90 mmHg |  |  |  |
| Heart rate (beats/min) >70 |  |  |  |
| Potassium |  |  |  |
| Hemoglobin |  |  |  |
| NT-proBNP |  |  |  |

Abbreviations: ICD-10, International Classification of Diseases, Tenth Revision codes; HF, heart failure; HFpEF, heart failure with preserved ejection fraction; HFmrEF, heart failure with mildly reduced ejection fraction; HFrEF, heart failure with reduced ejection fraction; CKD, chronic kidney disease; TIA, transient ischemic attack; COPD, Chronic obstructive pulmonary disease; RASi, renin–angiotensin system inhibitor; ARNI, angiotensin receptor neprilysin inhibitor; MRA, mineralocorticoid receptor antagonists; SGLT2i, sodium/glucose cotransporter-2 inhibitors, NT-proBNP, N-terminal pro-B-type natriuretic peptide; NYHA, New York Heart Association, BMI, body mass index.

**Table S2. Baseline characteristics among patients with new-onset HF stratified by KDIGO eGFR categories**

| **Characteristics** | | **HF** | | | | | |
| --- | --- | --- | --- | --- | --- | --- | --- |
|  | | **G2 (60–89)** | **G3a (45–59)** | **G3b (30–44)** | **G4 (15–29)** | **G5 (<15)** | ***p-value*** |
| N (%) | | 22647 (60) | 8428 (22) | 4741 (13) | 1474 (4) | 270 (1) |  |
| **Demographics** | | | | | | | |
| Sex Male, n (%)^a^ | | 14510 (64) | 4645 (55) | 2379 (50) | 723 (49) | 162 (60) | <0.001 |
| Age, years, median [IQR]^a^ | | 74 [66, 81] | 79 [73, 84] | 81 [75, 86] | 82 [76, 86] | 77 [69, 83] | <0.001 |
| **History and comorbidities** | | | | | | | |
| Alcohol, n (%) | | 670 (3) | 156 (2) | 79 (2) | 16 (1) | 8 (3) | <0.001 |
| Diabetes, n (%)^a^ | | 4037 (18) | 2028 (24) | 1460 (31) | 583 (40) | 105 (39) | <0.001 |
| Peripheral artery disease, n (%)^a^ | | 1356 (6) | 700 (8) | 502 (11) | 225 (15) | 59 (22) | <0.001 |
| Hypertension, n (%)^a^ | | 13528 (60) | 6026 (71) | 3688 (78) | 1242 (84) | 241 (89) | <0.001 |
| Stroke/TIA, n (%)^a^ | | 2884 (13) | 1426 (17) | 916 (19) | 355 (24) | 50 (19) | <0.001 |
| Ischemic heart disease, n (%)^a^ | | 9480 (42) | 4171 (49) | 2545 (54) | 845 (57) | 155 (57) | <0.001 |
| Atrial fibrillation, n (%)^a^ | | 11080 (49) | 4541 (54) | 2581 (54) | 716 (49) | 101 (37) | <0.001 |
| Malignant cancer within 3 years, n (%)^a^ | | 2692 (12) | 1129 (13) | 700 (15) | 246 (17) | 44 (16) | <0.001 |
| Valvular disease, n (%)^a^ | | 3611 (16) | 1611 (19) | 1018 (21) | 306 (21) | 51 (19) | <0.001 |
| COPD, n (%)^a^ | | 2217 (10) | 932 (11) | 569 (12) | 189 (13) | 37 (14) | <0.001 |
| Liver disease, n (%)^a^ | | 360 (2) | 105 (1) | 72 (2) | 17 (1) | 5 (2) | 0.17 |
| Musculoskeletal/connective tissue disease within 3 years, n (%)^a^ | | 6119 (27) | 2660 (32) | 1592 (34) | 541 (37) | 116 (43) | <0.001 |
| Dementia, n (%) | | 273 (1) | 134 (2) | 121 (3) | 31 (2) | 3 (1) | <0.001 |
| Depression, n (%) | | 762 (3) | 271 (3) | 180 (4) | 61 (4) | 8 (3) | 0.22 |
| CCI, n (%) | 0-1 | 8874 (39) | 2455 (29) | 901 (19) | 126 (9) | 7 (3) | <0.001 |
|  | 2-3 | 9031 (40) | 3446 (41) | 1752 (37) | 381 (26) | 51 (19) |  |
|  | 4-7 | 3926 (17) | 2110 (25) | 1740 (37) | 803 (54) | 160 (59) |  |
|  | >=8 | 816 (4) | 417 (5) | 348 (7) | 164 (11) | 52 (19) |  |
| **Medications** | | | | | | | |
| RASi/ARNI, n (%) | | 20779 (92) | 7462 (89) | 4001 (84) | 1067 (72) | 173 (64) | <0.001 |
| MRA, n (%) | | 8310 (37) | 3317 (39) | 1665 (35) | 358 (24) | 29 (11) | <0.001 |
| Digoxin, n (%) | | 3363 (15) | 1252 (15) | 604 (13) | 114 (8) | 10 (4) | <0.001 |
| Diuretic, n (%) | | 16236 (72) | 7096 (84) | 4331 (91) | 1378 (93) | 234 (87) | <0.001 |
| Nitrate, n (%) | | 5857 (26) | 2451 (29) | 1550 (33) | 518 (35) | 93 (34) | <0.001 |
| Platelet inhibitor, n (%) | | 10615 (47) | 4233 (50) | 2564 (54) | 872 (59) | 173 (64) | <0.001 |
| Statin, n (%) | | 11034 (49) | 4346 (52) | 2540 (54) | 774 (53) | 156 (58) | <0.001 |
| Oral anticoagulant, n (%) | | 10966 (48) | 4242 (50) | 2199 (46) | 591 (40) | 69 (26) | <0.001 |
| Beta-blockers, n (%) | | 20359 (90) | 7603 (90) | 4265 (90) | 1321 (90) | 238 (88) | 0.76 |
| Calcium channel blockers, n (%) | | 4810 (21) | 2401 (28) | 1614 (34) | 722 (49) | 177 (66) | <0.001 |
| SGLT2i, n (%) | | 552 (2) | 177 (2) | 85 (2) | 12 (1) | 0 (0) | <0.001 |
| **Socio-economic characteristics** | | | | | | | |
| Education, n (%)^a,b^ | Compulsory school | 9045 (40) | 3964 (47) | 2486 (52) | 803 (54) | 128 (47) | <0.001 |
|  | Secondary school | 9307 (41) | 3097 (37) | 1641 (35) | 499 (34) | 102 (38) |  |
|  | University | 4295 (19) | 1367 (16) | 614 (13) | 172 (12) | 40 (15) |  |
| Disposable income, Above median within year, n (%)^a,b^ | | 10977 (48) | 3159 (37) | 1496 (32) | 419 (28) | 98 (36) | <0.001 |
| Family type, Living alone, n (%)^a,b^ | | 10197 (45) | 4157 (49) | 2510 (53) | 855 (58) | 125 (46) | <0.001 |
| **Clinical characteristics** | | | | | | | |
| Location, In-patient, n (%) | | 9271 (41) | 4016 (48) | 2599 (55) | 976 (66) | 182 (67) | <0.001 |
| Follow-up referral, n (%) | Hospital | 16829 (77) | 5567 (69) | 2759 (62) | 775 (57) | 174 (70) | <0.001 |
|  | Primary care | 4487 (21) | 2274 (28) | 1533 (35) | 531 (39) | 56 (23) |  |
|  | Other | 529 (2) | 191 (2) | 146 (3) | 61 (4) | 18 (7) |  |
| EF category | HFrEF | 13098 (58) | 4429 (53) | 2291 (48) | 673 (46) | 119 (44) | <0.001 |
|  | HFmrEF | 5182 (23) | 1890 (22) | 1039 (22) | 309 (21) | 70 (26) |  |
|  | HFpEF | 4367 (19) | 2109 (25) | 1411 (30) | 492 (33) | 81 (30) |  |
| NYHA class, n (%) | I-II | 11301 (69) | 3438 (60) | 1622 (53) | 378 (45) | 65 (51) | <0.001 |
|  | III-IV | 5126 (31) | 2330 (40) | 1441 (47) | 462 (55) | 63 (49) |  |
| BMI (kg/m²) >=30, n (%) | | 3948 (24) | 1416 (24) | 845 (26) | 269 (28) | 46 (25) | 0.039 |
| Systolic blood pressure, (mmHg), median [IQR] | | 127 [114, 140] | 129 [115, 140] | 127 [114, 140] | 130 [116, 145] | 137 [120, 158] | <0.001 |
| Diastolic blood pressure, (mmHg), median [IQR] | | 75 [68, 81] | 72 [65, 80] | 70 [62, 80] | 70 [61, 80] | 74 [65, 80] | <0.001 |
| Mean arterial pressure >90 mmHg, n (%) | | 12221 (55) | 4315 (52) | 2244 (48) | 741 (51) | 159 (60) | <0.001 |
| Heart rate (beats/min) >70, n (%) | | 11947 (54) | 4511 (54) | 2537 (55) | 776 (54) | 141 (54) | 0.65 |
| Potassium (mmol/L), n (%) | Normakalemia | 18637 (95) | 6544 (92) | 3549 (89) | 1031 (86) | 180 (80) | <0.001 |
|  | Hypokalemia | 791 (4) | 378 (5) | 198 (5) | 71 (6) | 11 (5) |  |
|  | Hyperkalemia | 285 (1) | 215 (3) | 223 (6) | 102 (8) | 34 (15) |  |
| Hemoglobin, (g/L), median [IQR] | | 137 [125, 148] | 131 [120, 143] | 126 [114, 138] | 118 [107, 129] | 110 [102, 121] | <0.001 |
| NT-proBNP (pg/ml), Above median,n (%) | | 6693 (48) | 3096 (61) | 1956 (70) | 649 (82) | 127 (91) | <0.001 |

^a^ Variables included as covariates in the multivariable multinomial regression model.

KDIGO eGFR categories: G2 (60–89 mL/min/1.73 m², reference), G3a (45–59), G3b (30–44), G4 (15–29), and G5 (<15). Percentages are calculated within each KDIGO category (G2–G5). Patients with eGFR ≥90 mL/min/1.73 m² (n=11,889) were excluded from stratified analyses.

Hypokalaemia, normokalaemia, and hyperkalaemia were defined as serum potassium levels of <3.5, 3.5–5.0, and ≥5.0 mEq/L, respectively.

Abbreviations: HF, heart failure; eGFR, estimated glomerular filtration rate; HFrEF, heart failure with reduced ejection fraction; HFmrEF, heart failure with mildly reduced ejection fraction; HFpEF, heart failure with preserved ejection fraction; IQR, interquartile range; TIA, transient ischemic attack; COPD, chronic obstructive pulmonary disease; CCI, Charlson comorbidity index; RASi, renin-angiotensin system inhibitor; ARNI, angiotensin receptor neprilysin inhibitor; MRA, mineralocorticoid receptor antagonists; SGLT2i, sodium/glucose cotransporter-2 inhibitors; NT-proBNP, N-terminal pro-B-type natriuretic peptide; NYHA, New York Heart Association, BMI, body mass index.

**Table S3. Baseline characteristics among patients with new-onset HF across the EF spectrum stratified by KDIGO eGFR categories**

| **Characteristics** | | | **HFrEF** | | | | | | **HFmrEF** | | | | | | **HFpEF** | | | | | |
| --- | --- | --- | --- | --- | --- | --- | --- | --- | --- | --- | --- | --- | --- | --- | --- | --- | --- | --- | --- | --- |
|  | | | **G2 (60–89)** | **G3a (45–59)** | **G3b (30–44)** | **G4 (15–29)** | **G5 (<15)** | ***p-value*** | **G2 (60–89)** | **G3a (45–59)** | **G3b (30–44)** | **G4 (15–29)** | **G5 (<15)** | ***p-value*** | **G2 (60–89)** | **G3a (45–59)** | **G3b (30–44)** | **G4 (15–29)** | **G5 (<15)** | ***p-value*** |
| N (%) | | | 13098 (64) | 4429 (21) | 2291 (11) | 673 (3) | 119 (1) |  | 5182 (61) | 1890 (22) | 1039 (12) | 309 (4) | 70  (1) |  | 4367 (52) | 2109 (25) | 1411 (17) | 492 (6) | 81  (1) |  |
| **Demographics** | | | | | | | | | | | | | | | | | | | | |
| Sex Male, n (%)^a^ | | | 9210 (70) | 2779 (63) | 1318 (58) | 377 (56) | 74 (62) | <0.001 | 3254 (63) | 1006 (53) | 527 (51) | 159 (51) | 44 (63) | <0.001 | 2046 (47) | 860 (41) | 534 (38) | 187 (38) | 44 (54) | <0.001 |
| Age, years, median [IQR]^a^ | | | 72 [63, 79] | 77 [70, 83] | 80 [74, 85] | 81 [75, 86] | 76 [67, 82] | <0.001 | 75 [67, 81] | 79 [74, 84] | 81 [75, 85] | 82 [76, 86] | 78 [69, 82] | <0.001 | 79 [72, 84] | 81 [76, 85] | 82 [77, 87] | 83 [78, 87] | 80 [73, 84] | <0.001 |
| **History and comorbidities** | | | | | | | | | | | | | | | | | | | | |
| Alcohol, n (%) | | | 450 (3) | 99 (2) | 42 (2) | 11 (2) | 5  (4) | <0.001 | 122 (2) | 20  (1) | 16  (2) | 3  (1) | 3  (4) | 0.002 | 98  (2) | 37  (2) | 21  (1) | 2 (<1) | 0  (0) | 0.018 |
| Diabetes, n (%)^a^ | | | 2218 (17) | 1024 (23) | 656 (29) | 237 (35) | 43 (36) | <0.001 | 901 (17) | 448 (24) | 339 (33) | 131 (42) | 25 (36) | <0.001 | 918 (21) | 556 (26) | 465 (33) | 215 (44) | 37 (46) | <0.001 |
| Peripheral artery disease, n (%)^a^ | | | 696 (5) | 351 (8) | 251 (11) | 97 (14) | 24 (20) | <0.001 | 323 (6) | 163 (9) | 98  (9) | 41 (13) | 17 (24) | <0.001 | 337 (8) | 186 (9) | 153 (11) | 87 (18) | 18 (22) | <0.001 |
| Hypertension, n (%)^a^ | | | 7011 (54) | 2875 (65) | 1668 (73) | 533 (79) | 105 (88) | <0.001 | 3289 (63) | 1453 (77) | 812 (78) | 266 (86) | 60 (86) | <0.001 | 3228 (74) | 1698 (81) | 1208 (86) | 443 (90) | 76 (94) | <0.001 |
| Stroke/TIA, n (%)^a^ | | | 1477 (11) | 714 (16) | 419 (18) | 157 (23) | 19 (16) | <0.001 | 677 (13) | 325 (17) | 201 (19) | 76 (25) | 9  (13) | <0.001 | 730 (17) | 387 (18) | 296 (21) | 122 (25) | 22 (27) | <0.001 |
| Ischemic heart disease, n (%)^a^ | | | 5287 (40) | 2238 (51) | 1266 (55) | 409 (61) | 64 (54) | <0.001 | 2500 (48) | 1018 (54) | 595 (57) | 187 (61) | 42 (60) | <0.001 | 1693 (39) | 915 (43) | 684 (48) | 249 (51) | 49 (60) | <0.001 |
| Atrial fibrillation, n (%)^a^ | | | 5832 (45) | 2128 (48) | 1134 (49) | 299 (44) | 41 (34) | <0.001 | 2613 (50) | 1121 (59) | 585 (56) | 163 (53) | 27 (39) | <0.001 | 2635 (60) | 1292 (61) | 862 (61) | 254 (52) | 33 (41) | <0.001 |
| Malignant cancer within 3 years, n (%)^a^ | | | 1412 (11) | 557 (13) | 328 (14) | 115 (17) | 17 (14) | <0.001 | 679 (13) | 265 (14) | 153 (15) | 40 (13) | 10 (14) | 0.63 | 601 (14) | 307 (15) | 219 (16) | 91 (18) | 17 (21) | 0.017 |
| Valvular disease, n (%)^a^ | | | 1585 (12) | 679 (15) | 415 (18) | 121 (18) | 15 (13) | <0.001 | 901 (17) | 393 (21) | 235 (23) | 61 (20) | 16 (23) | <0.001 | 1125 (26) | 539 (26) | 368 (26) | 124 (25) | 20 (25) | 0.99 |
| COPD, n (%)^a^ | | | 1151 (9) | 453 (10) | 231 (10) | 72 (11) | 17 (14) | 0.004 | 492 (9) | 196 (10) | 137 (13) | 36 (12) | 7  (10) | 0.008 | 574 (13) | 283 (13) | 201 (14) | 81 (16) | 13 (16) | 0.27 |
| Liver disease, n (%)^a^ | | | 220 (2) | 45 (1) | 28 (1) | 7  (1) | 4  (3) | 0.005 | 54  (1) | 23  (1) | 18  (2) | 4  (1) | 1  (1) | 0.45 | 86  (2) | 37  (2) | 26  (2) | 6  (1) | 0  (0) | 0.55 |
| Musculoskeletal/connective tissue disease within 3 years, n (%)^a^ | | | 3042 (23) | 1236 (28) | 678 (30) | 238 (35) | 56 (47) | <0.001 | 1518 (29) | 662 (35) | 345 (33) | 107 (35) | 27 (39) | <0.001 | 1559 (36) | 762 (36) | 569 (40) | 196 (40) | 33 (41) | 0.013 |
| Dementia, n (%) | | | 122 (1) | 69 (2) | 55 (2) | 17 (3) | 0 (0) | <0.001 | 52  (1) | 23  (1) | 35  (3) | 3  (1) | 1  (1) | <0.001 | 99  (2) | 42  (2) | 31  (2) | 11  (2) | 2  (2) | 0.97 |
| Depression, n (%) | | | 446 (3) | 141 (3) | 79 (3) | 35 (5) | 3  (3) | 0.11 | 158 (3) | 55  (3) | 36  (3) | 9  (3) | 4  (6) | 0.67 | 158 (4) | 75  (4) | 65  (5) | 17  (3) | 1  (1) | 0.30 |
| CCI, n (%) | | 0-1 | 5456 (42) | 1300 (29) | 450 (20) | 54 (8) | 5  (4) | <0.001 | 1923 (37) | 554 (29) | 191 (18) | 25  (8) | 1  (1) | <0.001 | 1495 (34) | 601 (28) | 260 (18) | 47 (10) | 1  (1) | <0.001 |
|  | | 2-3 | 5161 (39) | 1869 (42) | 869 (38) | 177 (26) | 26 (22) |  | 2129 (41) | 740 (39) | 391 (38) | 84 (27) | 16 (23) |  | 1741 (40) | 837 (40) | 492 (35) | 120 (24) | 9  (11) |  |
|  | | 4-7 | 2065 (16) | 1058 (24) | 832 (36) | 367 (55) | 67 (56) |  | 925 (18) | 492 (26) | 376 (36) | 170 (55) | 44 (63) |  | 936 (21) | 560 (27) | 532 (38) | 266 (54) | 49 (60) |  |
|  | | >=8 | 416 (3) | 202 (5) | 140 (6) | 75 (11) | 21 (18) |  | 205 (4) | 104 (6) | 81  (8) | 30 (10) | 9  (13) |  | 195 (4) | 111 (5) | 127 (9) | 59 (12) | 22 (27) |  |
| **Medications** | | | | | | | | | | | | | | | | | | | | |
| RASi/ARNI, n (%) | | | 12565 (96) | 4146 (94) | 2051 (90) | 515 (77) | 87 (73) | <0.001 | 4756 (92) | 1657 (88) | 877 (84) | 240 (78) | 42 (60) | <0.001 | 3458 (79) | 1659 (79) | 1073 (76) | 312 (63) | 44 (54) | <0.001 |
| MRA, n (%) | | | 5535 (42) | 1904 (43) | 841 (37) | 171 (25) | 14 (12) | <0.001 | 1358 (26) | 613 (32) | 335 (32) | 47 (15) | 5  (7) | <0.001 | 1417 (32) | 800 (38) | 489 (35) | 140 (28) | 10 (12) | <0.001 |
| Digoxin, n (%) | | | 1948 (15) | 630 (14) | 299 (13) | 49 (7) | 4  (3) | <0.001 | 658 (13) | 275 (15) | 126 (12) | 30 (10) | 2  (3) | 0.006 | 757 (17) | 347 (16) | 179 (13) | 35  (7) | 4  (5) | <0.001 |
| Diuretic, n (%) | | | 9533 (73) | 3720 (84) | 2081 (91) | 625 (93) | 99 (83) | <0.001 | 3193 (62) | 1530 (81) | 930 (90) | 291 (94) | 62 (89) | <0.001 | 3510 (80) | 1846 (88) | 1320 (94) | 462 (94) | 73 (90) | <0.001 |
| Nitrate, n (%) | | | 3354 (26) | 1360 (31) | 793 (35) | 247 (37) | 38 (32) | <0.001 | 1562 (30) | 586 (31) | 343 (33) | 115 (37) | 24 (34) | 0.046 | 941 (22) | 505 (24) | 414 (29) | 156 (32) | 31 (38) | <0.001 |
| Platelet inhibitor, n (%) | | | 6285 (48) | 2370 (54) | 1323 (58) | 422 (63) | 73 (61) | <0.001 | 2554 (49) | 950 (50) | 569 (55) | 176 (57) | 41 (59) | 0.001 | 1776 (41) | 913 (43) | 672 (48) | 274 (56) | 59 (73) | <0.001 |
| Statin, n (%) | | | 6343 (48) | 2363 (53) | 1251 (55) | 359 (53) | 67 (56) | <0.001 | 2751 (53) | 994 (53) | 584 (56) | 175 (57) | 39 (56) | 0.25 | 1940 (44) | 989 (47) | 705 (50) | 240 (49) | 50 (62) | <0.001 |
| Oral anticoagulant, n (%) | | | 6173 (47) | 2097 (47) | 1024 (45) | 261 (39) | 33 (28) | <0.001 | 2459 (47) | 1019 (54) | 505 (49) | 130 (42) | 17 (24) | <0.001 | 2334 (53) | 1126 (53) | 670 (47) | 200 (41) | 19 (23) | <0.001 |
| Beta-blockers, n (%) | | | 12220 (93) | 4097 (93) | 2121 (93) | 616 (92) | 107 (90) | 0.092 | 4528 (87) | 1695 (90) | 939 (90) | 276 (89) | 65 (93) | 0.008 | 3611 (83) | 1811 (86) | 1205 (85) | 429 (87) | 66 (81) | 0.002 |
| Calcium channel blockers, n (%) | | | 2206 (17) | 966 (22) | 638 (28) | 263 (39) | 63 (53) | <0.001 | 1148 (22) | 587 (31) | 381 (37) | 170 (55) | 53 (76) | <0.001 | 1456 (33) | 848 (40) | 595 (42) | 289 (59) | 61 (75) | <0.001 |
| SGLT2i, n (%) | | | 409 (3) | 120 (3) | 53 (2) | 9  (1) | 0 (0) | 0.004 | 94  (2) | 34  (2) | 14  (1) | 1 (<1) | 0  (0) | 0.20 | 49  (1) | 23  (1) | 18  (1) | 2 (<1) | 0  (0) | 0.47 |
| **Socio-economic characteristics** | | | | | | | | | | | | | | | | | | | | |
| Education, n (%)^a^ | Compulsory school | | 5019 (38) | 2001 (45) | 1190 (52) | 358 (53) | 52 (44) | <0.001 | 2056 (40) | 923 (49) | 510 (49) | 168 (54) | 33 (47) | <0.001 | 1970 (45) | 1040 (49) | 786 (56) | 277 (56) | 43 (53) | <0.001 |
|  | Secondary school | | 5598 (43) | 1695 (38) | 801 (35) | 231 (34) | 48 (40) |  | 2084 (40) | 668 (35) | 380 (37) | 109 (35) | 25 (36) |  | 1625 (37) | 734 (35) | 460 (33) | 159 (32) | 29 (36) |  |
|  | University | | 2481 (19) | 733 (17) | 300 (13) | 84 (12) | 19 (16) |  | 1042 (20) | 299 (16) | 149 (14) | 32 (10) | 12 (17) |  | 772 (18) | 335 (16) | 165 (12) | 56 (11) | 9  (11) |  |
| Disposable income, Above median within year, n (%)^a^ | | | 6753 (52) | 1773 (40) | 753 (33) | 209 (31) | 41 (34) | <0.001 | 2541 (49) | 696 (37) | 353 (34) | 89 (29) | 24 (34) | <0.001 | 1683 (39) | 690 (33) | 390 (28) | 121 (25) | 33 (41) | <0.001 |
| Family type, Living alone, n (%)^a^ | | | 5758 (44) | 2111 (48) | 1150 (50) | 383 (57) | 57 (48) | <0.001 | 2197 (42) | 912 (48) | 536 (52) | 167 (54) | 31 (44) | <0.001 | 2242 (51) | 1134 (54) | 824 (58) | 305 (62) | 37 (46) | <0.001 |
| **Clinical characteristics** | | | | | | | | | | | | | | | | | | | | |
| Location, In-patient, n (%) | | | 5009 (38) | 1933 (44) | 1127 (49) | 413 (61) | 66 (55) | <0.001 | 1862 (36) | 851 (45) | 548 (53) | 190 (61) | 48 (69) | <0.001 | 2400 (55) | 1232 (58) | 924 (65) | 373 (76) | 68 (84) | <0.001 |
| Follow-up referral, n (%) | | Hospital | 10846 (85) | 3390 (79) | 1539 (71) | 409 (65) | 82 (74) | <0.001 | 3715 (74) | 1152 (64) | 589 (61) | 159 (55) | 53 (82) | <0.001 | 2268 (55) | 1025 (52) | 631 (49) | 207 (46) | 39 (54) | <0.001 |
|  | | Primary care | 1602 (13) | 782 (18) | 571 (26) | 197 (31) | 20 (18) |  | 1188 (24) | 592 (33) | 346 (36) | 117 (41) | 9  (14) |  | 1697 (41) | 900 (46) | 616 (47) | 217 (48) | 27 (38) |  |
|  | | Other | 267 (2) | 98 (2) | 60 (3) | 23 (4) | 9  (8) |  | 106 (2) | 43  (2) | 33  (3) | 11  (4) | 3  (5) |  | 156 (4) | 50  (3) | 53  (4) | 27  (6) | 6  (8) |  |
| NYHA class, n (%) | | I-II | 6670 (67) | 1841 (56) | 826 (50) | 162 (38) | 26 (41) | <0.001 | 2907 (76) | 852 (67) | 398 (59) | 80 (46) | 22 (65) | <0.001 | 1724 (66) | 745 (60) | 398 (54) | 136 (56) | 17 (57) | <0.001 |
|  | | III-IV | 3331 (33) | 1418 (44) | 836 (50) | 259 (62) | 38 (59) |  | 911 (24) | 425 (33) | 271 (41) | 95 (54) | 12 (35) |  | 884 (34) | 487 (40) | 334 (46) | 108 (44) | 13 (43) |  |
| BMI (kg/m²) >=30, n (%) | | | 2199 (23) | 692 (22) | 335 (21) | 105 (23) | 18 (20) | 0.59 | 909 (25) | 296 (23) | 205 (28) | 55 (27) | 14 (30) | 0.075 | 840 (29) | 428 (30) | 305 (32) | 109 (34) | 14 (29) | 0.30 |
| Systolic blood pressure, (mmHg), median [IQR] | | | 123 [110, 140] | 124 [110, 140] | 122 [110, 140] | 125 [110, 140] | 129 [110, 146] | 0.072 | 130 [120, 144] | 130 [120, 145] | 130 [116, 141] | 130 [120, 149] | 140 [125, 156] | <0.001 | 130 [120, 148] | 132 [120, 150] | 130 [119, 150] | 135 [120, 150] | 148 [129, 165] | <0.001 |
| Diastolic blood pressure, (mmHg), median [IQR] | | | 75 [67, 82] | 72 [65, 80] | 70 [62, 80] | 70 [61, 80] | 70 [60, 80] | <0.001 | 75 [70, 81] | 75 [65, 80] | 70 [64, 80] | 70 [62, 80] | 75 [66, 81] | <0.001 | 75 [66, 80] | 72 [65, 80] | 70 [62, 80] | 70 [60, 80] | 75 [68, 80] | <0.001 |
| Mean arterial pressure >90 mmHg, n (%) | | | 6645 (51) | 2073 (47) | 990 (44) | 311 (47) | 58 (50) | <0.001 | 3003 (59) | 1053 (57) | 520 (51) | 157 (52) | 44 (65) | <0.001 | 2573 (60) | 1189 (57) | 734 (53) | 273 (56) | 57 (71) | <0.001 |
| Heart rate (beats/min) >70, n (%) | | | 7136 (55) | 2433 (56) | 1284 (57) | 382 (58) | 68 (59) | 0.34 | 2557 (50) | 987 (54) | 534 (53) | 153 (50) | 40 (59) | 0.10 | 2254 (53) | 1091 (53) | 719 (52) | 241 (50) | 33 (42) | 0.27 |
| Potassium (mmol/L), n (%) | | Normakalemia | 10904 (95) | 3489 (92) | 1701 (89) | 461 (84) | 77 (75) | <0.001 | 4336 (95) | 1475 (92) | 816 (90) | 216 (85) | 51 (85) | <0.001 | 3397 (92) | 1580 (90) | 1032 (89) | 354 (88) | 52 (83) | <0.001 |
|  | | Hypokalemia | 368 (3) | 173 (5) | 78 (4) | 29 (5) | 5  (5) |  | 178 (4) | 84  (5) | 42  (5) | 17  (7) | 4  (7) |  | 245 (7) | 121 (7) | 78  (7) | 25  (6) | 2  (3) |  |
|  | | Hyperkalemia | 193 (2) | 124 (3) | 122 (6) | 57 (10) | 20 (20) |  | 54  (1) | 45  (3) | 48  (5) | 21  (8) | 5  (8) |  | 38  (1) | 46  (3) | 53  (5) | 24  (6) | 9  (14) |  |
| Hemoglobin, (g/L), median [IQR] | | | 139 [127, 150] | 134 [122, 146] | 129 [116, 141] | 120 [109, 131] | 113 [102, 124] | <0.001 | 136 [124, 147] | 130 [118, 142] | 124 [114, 137] | 117 [105, 127] | 108 [102, 117] | <0.001 | 130 [119, 141] | 127 [116, 138] | 122 [111, 134] | 117 [106, 128] | 110 [102, 120] | <0.001 |
| NT-proBNP (pg/ml), Above median,n (%) | | | 4464 (54) | 1825 (68) | 1039 (78) | 325 (89) | 56 (92) | <0.001 | 1190 (38) | 613 (55) | 433 (67) | 132 (80) | 33 (92) | <0.001 | 1039 (39) | 658 (51) | 484 (59) | 192 (74) | 38 (88) | <0.001 |

^a^ Variables included as covariates in the multivariable multinomial regression model.

KDIGO eGFR categories: G2 (60–89 mL/min/1.73 m², reference), G3a (45–59), G3b (30–44), G4 (15–29), and G5 (<15). Percentages are calculated within each KDIGO category (G2–G5). Patients with eGFR ≥90 mL/min/1.73 m² (n=11,889) were excluded from stratified analyses.

Hypokalaemia, normokalaemia, and hyperkalaemia were defined as serum potassium levels of <3.5, 3.5–5.0, and ≥5.0 mEq/L, respectively.

Abbreviations: HFrEF, heart failure with reduced ejection fraction; HFmrEF, heart failure with mildly reduced ejection fraction; HFpEF, heart failure with preserved ejection fraction; IQR, interquartile range; TIA, transient ischemic attack; COPD, chronic obstructive pulmonary disease; CCI, Charlson comorbidity index; RASi, renin-angiotensin system inhibitor; ARNI, angiotensin receptor neprilysin inhibitor; MRA, mineralocorticoid receptor antagonists; SGLT2i, sodium/glucose cotransporter-2 inhibitors; NT-proBNP, N-terminal pro-B-type natriuretic peptide; NYHA, New York Heart Association, BMI, body mass index.

**Table S4. Interaction analyses between patient characteristics and CKD status for HF and HF phenotypes**

|  | **HF vs. non-HF** | | | **HFmrEF vs. HFrEF** | | | **HFpEF vs. HFrEF** | | |
| --- | --- | --- | --- | --- | --- | --- | --- | --- | --- |
|  | **no CKD**  (OR 95% CI**)** | **CKD**  (OR 95% CI**)** | ***p-value*** | **no CKD**  (OR 95% CI**)** | **CKD**  (OR 95% CI**)** | ***p-value*** | **no CKD**  (OR 95% CI**)** | **CKD**  (OR 95% CI**)** | ***p-value*** |
| Diabetes | 1.48 (1.38-1.58) | 1.07 (0.84-1.35) | 0.008 | 0.89 (0.84-0.94) | 1.48 (1.22-1.79) | <0.001 | 1.07 (1.01-1.13) | 1.70 (1.43-2.03) | <0.001 |
| Peripheral artery disease | 1.16 (1.03-1.29) | 0.95 (0.68-1.33) | 0.276 | 0.92 (0.84-1.01) | 0.90 (0.70-1.17) | 0.909 | 1.00 (0.91-1.09) | 0.87 (0.69-1.11) | 0.300 |
| Hypertension | 4.19 (3.99-4.40) | 1.86 (1.42-2.44) | <0.001 | 1.41 (1.34-1.48) | 1.48 (1.14-1.91) | 0.727 | 2.28 (2.16-2.41) | 2.52 (1.92-3.32) | 0.484 |
| Stroke/TIA | 0.64 (0.60-0.69) | 0.57 (0.43-0.76) | 0.465 | 1.01 (0.94-1.08) | 1.03 (0.81-1.31) | 0.854 | 1.17 (1.09-1.25) | 1.10 (0.89-1.36) | 0.602 |
| Ischemic heart disease | 5.34 (5.06-5.63) | 4.20 (3.28-5.36) | 0.058 | 1.28 (1.23-1.34) | 1.23 (1.02-1.49) | 0.666 | 0.81 (0.77-0.85) | 0.78 (0.66-0.93) | 0.717 |
| Atrial fibrillation | 13.56 (12.73-14.45) | 6.51 (4.96-8.54) | <0.001 | 1.23 (1.17-1.28) | 1.18 (0.97-1.42) | 0.676 | 1.61 (1.53-1.69) | 1.15 (0.97-1.37) | <0.001 |
| Malignant cancer within 3 years | 1.10 (1.03-1.18) | 0.65 (0.49-0.87) | <0.001 | 1.17 (1.10-1.26) | 0.95 (0.72-1.24) | 0.126 | 1.29 (1.20-1.38) | 1.31 (1.04-1.66) | 0.858 |
| Valvular disease | 6.53 (5.92-7.20) | 3.78 (2.54-5.62) | 0.008 | 1.42 (1.34-1.51) | 1.23 (0.96-1.58) | 0.265 | 2.17 (2.05-2.31) | 1.67 (1.34-2.08) | 0.022 |
| COPD | 3.14 (2.85-3.46) | 1.28 (0.85-1.92) | <0.001 | 1.04 (0.96-1.12) | 1.03 (0.76-1.39) | 0.959 | 1.44 (1.34-1.55) | 1.35 (1.04-1.75) | 0.626 |
| Musculoskeletal/connective tissue disease within 3 years | 1.11 (1.06-1.17) | 0.76 (0.60-0.97) | 0.002 | 1.28 (1.22-1.34) | 1.13 (0.93-1.37) | 0.220 | 1.55 (1.48-1.64) | 1.20 (1.01-1.44) | 0.007 |
| Liver disease | 1.83 (1.51-2.21) | 1.15 (0.55-2.41) | 0.233 | 0.81 (0.68-0.97) | 0.82 (0.48-1.40) | 0.986 | 1.05 (0.89-1.24) | 0.83 (0.51-1.36) | 0.370 |
| Secondary school vs. compulsory school | 0.96 (0.92-1.01) | 1.22 (0.94-1.58) | 0.076 | - | - | - | - | - | - |
| University vs. compulsory school | 0.81 (0.76-0.86) | 1.06 (0.77-1.47) | 0.095 | 1.10 (1.03-1.17) | 0.98 (0.75-1.29) | 0.432 | 1.01 (0.94-1.09) | 0.99 (0.77-1.28) | 0.889 |
| Income above vs. below median | 0.85 (0.81-0.89) | 0.77 (0.61-0.96) | 0.385 | 0.98 (0.93-1.02) | 0.81 (0.67-0.98) | 0.062 | 0.66 (0.63-0.70) | 0.77 (0.65-0.92) | 0.096 |
| Living alone vs. cohabiting | 1.21 (1.16-1.26) | 1.33 (1.06-1.68) | 0.405 | 0.94 (0.90-0.98) | 1.03 (0.85-1.24) | 0.366 | 1.24 (1.18-1.30) | 1.14 (0.96-1.36) | 0.350 |

Only patient characteristics significantly associated with new-onset HF in the conditional logistic regression models were subsequently tested in the multinomial regression models to assess whether their associations differed across EF phenotypes.

Abbreviations: HF, heart failure; CKD, chronic kidney disease; HFrEF, heart failure with reduced ejection fraction; HFmrEF, heart failure with mildly reduced ejection fraction; HFpEF, heart failure with preserved ejection fraction; OR, odds ratio; CI, confidence interval; TIA, transient ischemic attack; COPD, chronic obstructive pulmonary disease.

**Figure S1. Flow chart of the study population**

Abbreviations: HF, heart failure; HFpEF, heart failure with preserved ejection fraction; HFmrEF, heart failure with mildly reduced ejection fraction; HFrEF, heart failure with reduced ejection fraction; CKD, chronic kidney disease; SwedeHF, Swedish Heart Failure Registry; EF, ejection fraction; eGFR, estimated glomerular filtration rate; ICD-10, International Statistical Classification of Diseases and Related Health Problems, 10th Revision.

**Figure S2. Patient characteristics associated with new-onset HFmrEF vs. HFrEF onset according to CKD status**

**
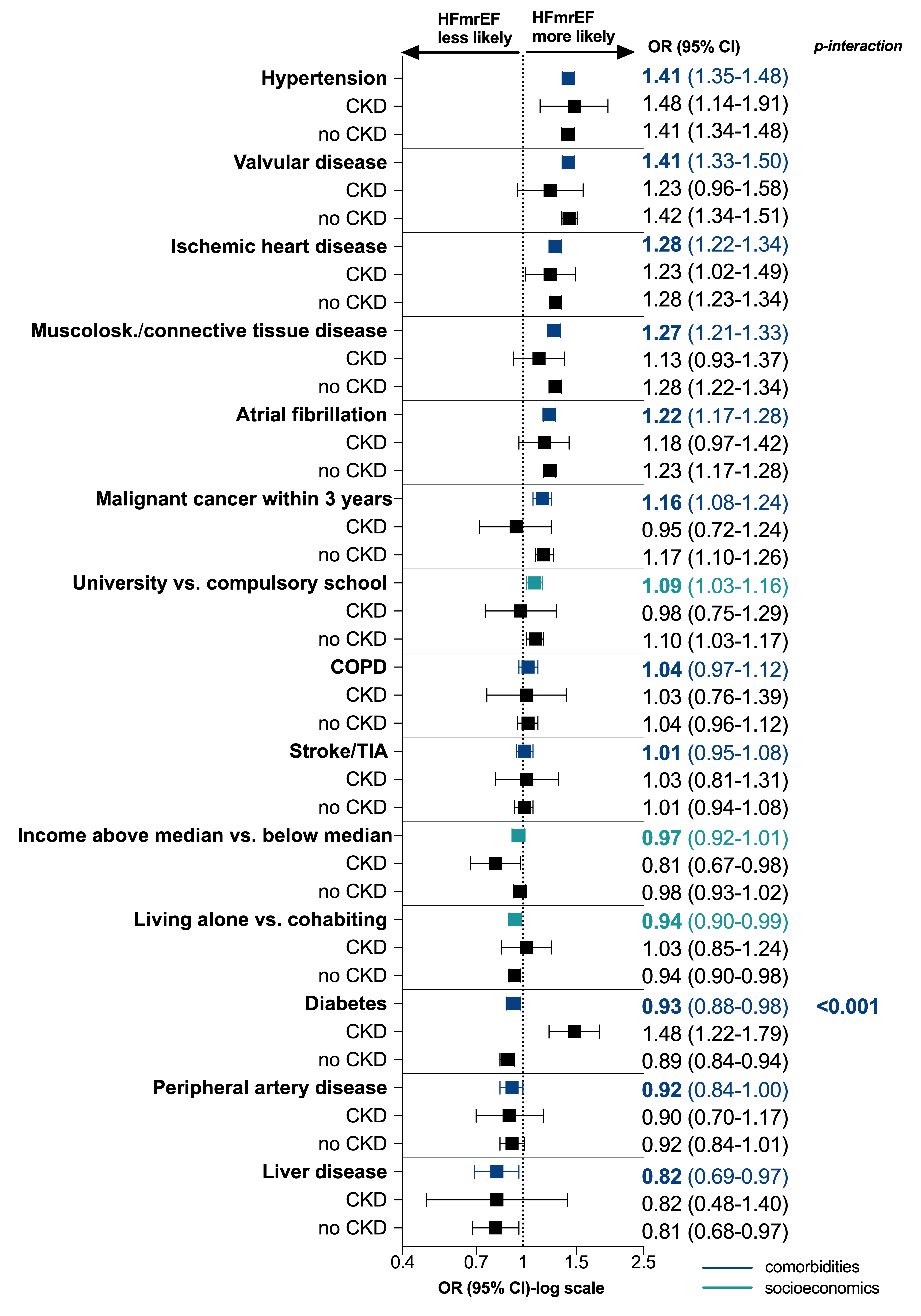
**

All covariates were tested for interaction with CKD; only significant results are displayed in the graph. Reference category: HFrEF.

Abbreviations: HFmrEF, heart failure with mildly reduced ejection fraction; CKD, chronic kidney disease; OR, odds ratio; CI, confidence intervals; p, p-value; COPD, chronic obstructive pulmonary disease; TIA, transient ischemic attack, muscolosk./connective tissue disease, musculoskeletal or connective tissue disease.

**Figure S3. Patient characteristics associated with new-onset HFpEF vs. HFrEF onset according to CKD status**


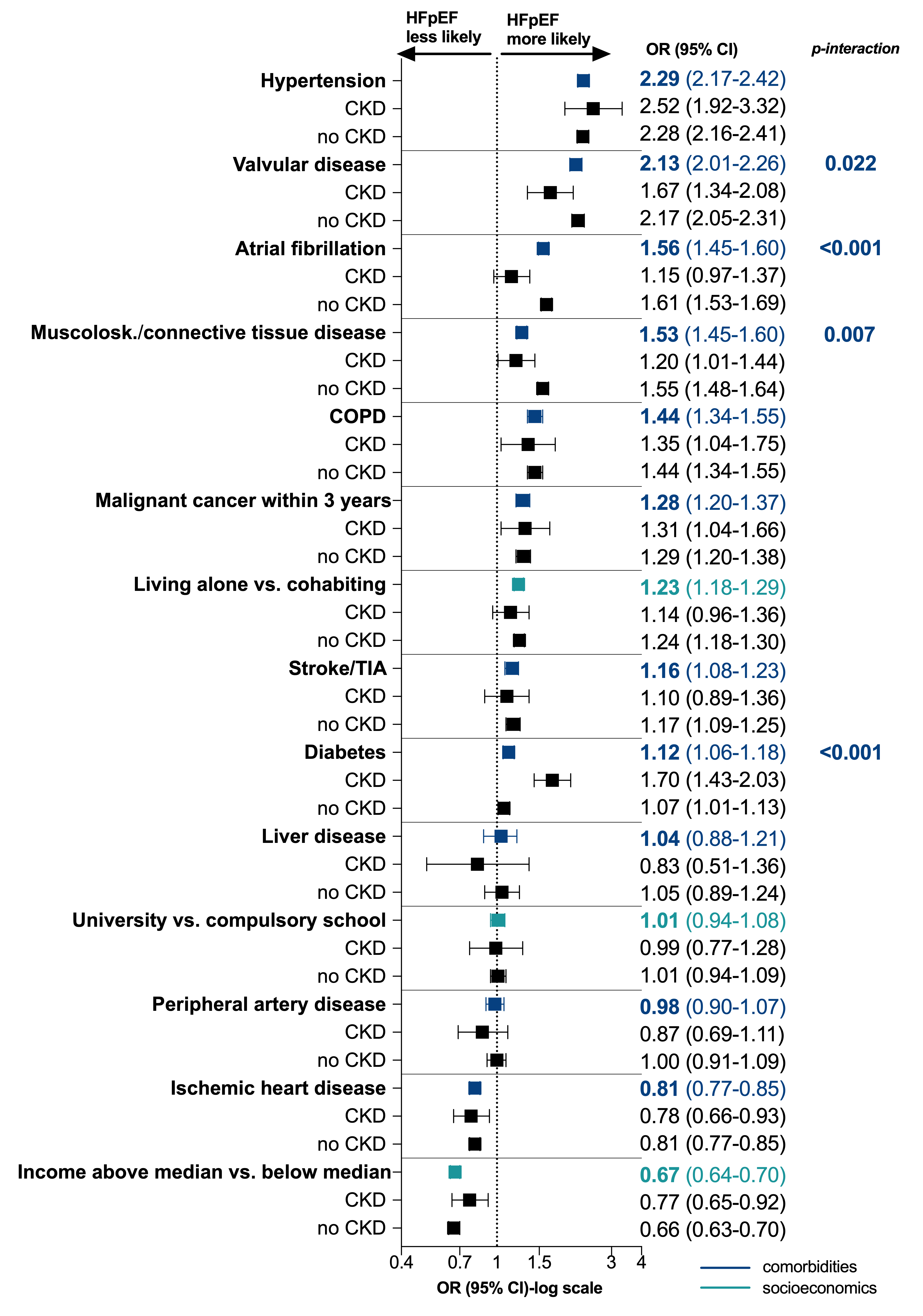


All covariates were tested for interaction with CKD; only significant results are displayed in the graph. Reference category: HFrEF.

Abbreviations: HFpEF, heart failure with preserved ejection fraction; CKD, chronic kidney disease; OR, odds ratio; CI, confidence intervals; p, p-value; COPD, chronic obstructive pulmonary disease; TIA, transient ischemic attack, muscolosk./connective tissue disease, musculoskeletal or connective tissue disease.

**References**

1. Ter Maaten JM, Damman K, Verhaar MC, et al. Connecting heart failure with preserved ejection fraction and renal dysfunction: the role of endothelial dysfunction and inflammation. *Eur J Heart Fail*. Jun 2016;18(6):588-98. doi:10.1002/ejhf.497

2. Narayanan G, Halim A, Hu A, et al. Molecular Phenotyping and Mechanisms of Myocardial Fibrosis in Advanced Chronic Kidney Disease. *Kidney360*. 2023;4(11):1562-1579. doi:10.34067/kid.0000000000000276

3. Junho CVC, Frisch J, Soppert J, Wollenhaupt J, Noels H. Cardiomyopathy in chronic kidney disease: clinical features, biomarkers and the contribution of murine models in understanding pathophysiology. *Clinical Kidney Journal*. 2023;16(11):1786-1803. doi:10.1093/ckj/sfad085

4. SwedeHF database 4 (SHFDB4). <https://kiheartfailure.github.io/shfdb4/definitions/>

5. Friberg L, Gasparini A, Carrero JJ. A scheme based on ICD-10 diagnoses and drug prescriptions to stage chronic kidney disease severity in healthcare administrative records. *Clin Kidney J*. Apr 2018;11(2):254-258. doi:10.1093/ckj/sfx085

6. Centers for Disease Control and Prevention- CKD Surveillance System: Methods. <https://nccd.cdc.gov/ckd/Methods.aspx?Qnum=Q637>

7. Savarese G, Kishi T, Vardeny O, et al. Heart Failure Drug Treatment—Inertia, Titration, and Discontinuation. *JACC: Heart Failure*. 2023;11(1):1-14. doi:doi:10.1016/j.jchf.2022.08.009

8. Sundström J, Ärnlöv J, Karayiannides S, et al. Heart failure outcomes by left ventricular ejection fraction in a contemporary region-wide patient cohort. *ESC Heart Fail*. Jun 2024;11(3):1377-1388. doi:10.1002/ehf2.14685

9. Ludvigsson JF, Appelros P, Askling J, et al. Adaptation of the Charlson Comorbidity Index for Register-Based Research in Sweden. *Clin Epidemiol*. 2021;13:21-41. doi:10.2147/clep.s282475

10. Sandhu AT, Tisdale RL, Rodriguez F, et al. Disparity in the Setting of Incident Heart Failure Diagnosis. *Circulation: Heart Failure*. 2021;14(8):e008538. doi:doi:10.1161/CIRCHEARTFAILURE.121.008538

11. Geng T, Li X, Ma H, Heianza Y, Qi L. Adherence to a Healthy Sleep Pattern and Risk of Chronic Kidney Disease: The UK Biobank Study. *Mayo Clin Proc*. Jan 2022;97(1):68-77. doi:10.1016/j.mayocp.2021.08.028

12.  United States Renal Data System U.S. Department of Health and Human Services- National Institutes of Health- CKD Analytical Methods. <https://usrds-adr.niddk.nih.gov/2022/chronic-kidney-disease/ckd-analytical-methods>
